# Assessment of precision irradiation in early non-small cell lung cancer and interstitial lung disease (ASPIRE-ILD): study protocol for a phase II trial

**DOI:** 10.1186/s12885-019-6392-8

**Published:** 2019-12-11

**Authors:** David A. Palma, Hanbo Chen, Houda Bahig, Stewart Gaede, Stephen Harrow, Joanna M. Laba, X. Melody Qu, George B. Rodrigues, Brian P. Yaremko, Edward Yu, Alexander V. Louie, Inderdeep Dhaliwal, Christopher J. Ryerson

**Affiliations:** 1 Department of Oncology, Western University, 1151 Richmond Street, London, Ontario N6A 3K7 Canada; 2 Department of Radiation Oncology, Centre Hospitalier de l’Université de Montréal, 1051 Sanguinet Street, Montreal, Quebec H2X 3E4 Canada; 3 Department of Clinical Oncology, Beatson West of Scotland Cancer Centre, 1053 Great Western Road, Glasgow, G12 0YN UK; 4 Department of Radiation Oncology, Sunnybrook Cancer Centre, 2075 Bayview Avenue, Toronto, Ontario M4N 3M5 Canada; 5 Department of Respirology, Western University, 1151 Richmond Street, London, Ontario N6A 3K7 Canada; 6 Department of Medicine, University of British Columbia, 2775 Laurel Street, Vancouver, British Columbia V5Z 1M9 Canada

**Keywords:** Stereotactic radiotherapy, Cancer, Lung, Interstitial lung disease, Toxicity, Survival

## Abstract

**Background:**

Stereotactic ablative radiotherapy (SABR) has become an established treatment option for medically-inoperable early-stage (Stage I-IIA) non-small cell lung cancer (ES-NSCLC). SABR is able to obtain high rates of local control with low rates of symptomatic toxicity in this patient population. However, in a subset of patients with fibrotic interstitial lung disease (ILD), elevated rates of SABR-related toxicity and mortality have been described. The Assessment of Precision Irradiation in Early Non-Small Cell Lung Cancer and Interstitial Lung Disease (ASPIRE-ILD) study will conduct a thorough prospective evaluation of the clinical outcomes, toxicity, changes in diagnostic test parameters and patient-related outcomes following SABR for ES-NSCLC for patients with fibrotic ILD.

**Methods:**

ASPIRE-ILD is a single-arm Phase II prospective study. The accrual target is 39 adult patients with T1–2N0M0 non-small cell lung cancer with co-existing ILD who are not candidates for surgical excision. Pathological confirmation of diagnosis is strongly recommended but not strictly required. Enrolled patients will be stratified by ILD-related mortality risk. The starting SABR dose will be 50 Gy in 5 fractions every other day (biologically effective dose: 100 Gy_10_ or 217 Gy_3_), but the radiation dose can be de-escalated up to two times to 50 Gy in 10 fractions daily (75 Gy_10_ or 133 Gy_3_) and 45 Gy in 15 fractions daily (58 Gy_10_ or 90 Gy_3_). Dose de-escalation will occur if 2 or more of the first 7 patients in a cohort experiences grade 5 toxicity within 6 months of treatment. Similarly, dose de-escalation can also occur if 2 or more of the first 7 patients with a specific subtype of ILD experiences grade 5 toxicity within 6 months of treatment. The primary endpoint is overall survival. Secondary endpoints include toxicity (CTC-AE 4.0), progression-free survival, local control, patient-reported outcomes (cough severity and quality of life), rates of ILD exacerbation and changes in pulmonary function tests/high-resolution computed tomography findings post-SABR.

**Discussion:**

ASPIRE-ILD will be the first prospective study specifically designed to comprehensively evaluate the effectiveness and safety of SABR for ES-NSCLC in patients with co-existing ILD.

**Trial registration:**

Clinicaltrials.gov identifier: NCT03485378. Date of registration: April 2, 2018.

## Background

Non-small cell lung cancer (NSCLC) is the leading cause of cancer death in men and women worldwide. Approximately 20% of NSCLC patients present with early stage disease (T1 N0 or T2 N0), defined as tumors up to 5 cm in size, without nodal or distant metastases. For such patients, surgical resection has been considered the standard of care; however, many patients are ineligible for surgery because of co-morbid conditions.

Historically, alternatives to surgery have proven unsatisfactory. Most patients who were not candidates for surgery were treated with conventional radiotherapy, delivered as doses of 50–60 Gy in 4–6 weeks, with relatively rudimentary tumor targeting techniques. Conventional radiotherapy was associated with high rates of local recurrence, often 30–40% or higher, with only a minor improvement in long-term survival compared to observation alone [1, 2].

Stereotactic ablative radiotherapy (SABR) is a newer radiotherapy approach which uses modern radiotherapy planning and targeting technologies to precisely deliver larger, ablative doses of radiotherapy (up to 60 Gy in 3–8 fractions). SABR has been associated with high rates of local control, with many studies reporting 3-year local control of approximately 90% after SABR, comparable to results obtained with anatomic lobectomy [3]. Because of these promising outcomes, randomized trials are currently comparing SABR vs. surgery as first-line treatment for early-stage NSCLC (ES-NSCLC).

A major advantage of SABR is that in general, the toxicity profile is very favorable, even in patients with substantial co-morbid conditions. Radiation Therapy Oncology Group (RTOG) 0236, a North American phase II trial assessing outcomes with SABR for ES-NSCLC in patients who were unfit for surgery, demonstrated a risk of protocol-specified Grade 3 and 4 toxicities of 13 and 4% respectively, with no grade 5 toxicities [4]. Based on a large meta-analysis, the risk of treatment related death after SABR is estimated at < 1%. By comparison, after anatomic lobectomy, toxicity rates appear higher: with Grade 3 or 4 toxicity often approaching 50%, and 30-day mortality rates of 4–6% based on Medicare data, although this latter risk is lower at specialized high-volume centers [5, 6].

### Interstitial lung disease and SABR

Interstitial lung disease (ILD), also known as diffuse parenchymal lung disease, comprises a heterogeneous group of diseases characterized by a non-neoplastic, diffuse inflammatory and/or fibrotic pathology affecting the lung parenchyma, usually exhibiting a restrictive defect on pulmonary function testing with reduced gas exchange [7, 8].

ILD can be subdivided into fibrotic and non-fibrotic subtypes. Fibrotic subtypes include idiopathic pulmonary fibrosis (IPF), connective tissue disease-associated ILD (CTD-ILD), idiopathic non-specific interstitial pneumonia, hypersensitivity pneumonitis, and unclassifiable ILD. Fibrotic ILDs are characterized by reticulation, traction bronchiectasis, and frequently honeycombing on high-resolution computed tomography (HRCT). These fibrotic subtypes of ILD are the focus on this research protocol.

Patients with NSCLC and co-existing fibrotic ILD represent a high-risk group for any type of cancer treatment. Patients with ILD and NSCLC are at risk of both treatment-related toxicities and acute exacerbation of ILD [9], which can be life-threatening and even fatal. Despite the generally favorable toxicity profile of SABR, emerging data indicate that patients with pre-existing ILD are at particularly high risk of toxicity after SABR. A recent meta-analysis examined outcomes after several different treatments for early-stage lung cancer in patients with ILD [10]. Within the meta-analysis, 13 published studies (Additional file 1) assessed outcomes after SABR. Overall, the use of SABR in patients with early-stage NSCLC and pre-existing ILD was associated with a 25% risk of radiation pneumonitis (grade 3 or higher) and a 15% risk of treatment-related death. The risk may be highest in the subset of patients with IPF: SABR-related mortality was 33% in studies limited to IPF patients, and 14% in other studies that included only non-IPF fibrotic ILD, or a combination of IPF and non-IPF fibrotic ILD patients (*p* = 0.092).

However, the true rates of toxicity likely differ from the results of this meta-analysis, as nearly all the included studies were retrospective, a wide range of doses were used, and there was substantial variability in outcomes (e.g., treatment-related death ranged from 0 to 60% across studies). There is also risk of publication bias favoring the publication studies that show remarkable results (i.e. high rates of toxicity). Compounding these limitations, attribution of toxicity to radiation is difficult, since ILD exacerbations and radiation pneumonitis may result in similar clinical presentations.

Therefore, the true rates of toxicity of SABR in fibrotic ILD patients are likely higher than in the general population, but the precise extent of toxicity remains unknown.

### ILD severity

Even in the absence of lung cancer, the natural history of ILD is variable. Some patients survive many years with only a modest decline in lung function, whereas other patients experience rapid respiratory decline and death within a few short months.

The ILD-GAP (Gender-Age-Physiology) system (Table 1) is a prognostic model that takes into account disease type and severity, age, and gender, to predict survival in patients with the major subtypes of chronic ILD, including IPF [11]. The ILD-GAP index assigns an overall score between 0 and 8, with the majority of points assigned based on current pulmonary function. 3-year mortality ranges from 10% in patients with an index of 0–1 to 75% in patients with an index > 5. Notably, all patients with IPF or unclassifiable ILD have a minimum index of 2 in the ILD-GAP model, reflecting their inferior prognosis. We hypothesize that ILD subtype and severity of pulmonary impairment both influence the risk of radiation toxicity.

**Table 1:** The ILD-GAP model, reproduced from Ryerson et al. [11]

Predictor		Points
ILD	ILD Subtype	
	• Idiopathic pulmonary fibrosis	0
	• Unclassifiable ILD	0
	• Connective tissue-ILD / idiopathic non-specific interstitial pneumonia	−2
	• Chronic hypersensitivity pneumonitis	−2
G	Gender	
	• Female	0
	• Male	1
A	Age	
	• ≤ 60	0
	• 61–65	1
	• > 65	2
P	Physiology	
	FVC, % predicted	
	• > 75%	0
	• 50–75%	1
	• < 50%	2
	DLCO, % predicted	
	• > 55%	0
	• 36–55%	1
	• ≤ 35%	2
	• Cannot perform	3
Total Possible Points		8

### Rationale for a study

For patients with ILD and concurrent early-stage lung cancer who are not candidates for surgery, these data showing high rates of toxicity have led to a difficult clinical dilemma, since there are few alternate treatment options. The option of delivering no treatment whatsoever, which avoids any risk of treatment-related toxicity, is associated with a high risk of death due to the lung cancer itself. The median survival for untreated stage I NSCLC is only 6–8 months, with 1-year mortality of 60–70% [12], much higher than the risks associated with SABR treatment. For example, in one study of untreated patients with NSCLC in the U.S. National Cancer Database, the median survival for patients with stage I NSCLC who were unfit for an operation was 7.6 months [13]. This suggests that even in patients with an ILD-GAP score > 5, the risk of death due to lung cancer within 1 year exceeds the risk of death due to ILD or complications of SABR.

As a result, patients with ILD and early-stage NSCLC are often advised, after discussion of their case at a multidisciplinary conference, that treatment with SABR provides the best chance of local control and cure, but that SABR is associated with an appreciable risk of treatment-related toxicity. For patients undergoing SABR under this scenario, the optimal dose and fractionation is unknown.

It is possible that currently-used doses and fractionations of SABR, when given with strict planning criteria to minimize the risk of lung toxicity, have only a modest risk of treatment-related toxicity and represent the best possible approach.

Alternatively, it may be necessary to reduce the dose of SABR, at the risk of compromising tumor control. Adjustments to the SABR fractionation schedule may reduce the risk of toxicity, while still maintaining some benefit. Radiation dose is expressed in Gray (Gy), with most SABR treatments delivering approximately a total dose 50–60 Gy. This total dose is divided up into smaller individual fractions of radiation. Conventional radiotherapy employs fraction sizes of approximately 2 Gy per day, 5 days per week, and would require 30 fractions to deliver 60 Gy. This prolonged treatment time (i.e. 6 weeks of treatment) may allow for tumor repopulation, compromising treatment outcomes. With SABR, common fractionations include 54 Gy in 3 fractions (i.e. 18 Gy per fraction), 48 Gy in 4 fractions (i.e. 12 Gy per fraction) and 60 Gy in 8 fractions (i.e. 7.5 Gy per day). SABR fractions are often given every second day (or even more widely spaced) to maximize the time for normal tissue repair between fractions.

To compare the potency of these different radiation dose fractionations, a formula called the biologically effective dose (BED) is used. BED takes into account the total number of fractions, the dose per fraction, and the intrinsic radiation response curve of the individual tissues treated. BED is calculated according to the formula ${BED}_{\alpha /\beta }= nd\left(1+\frac{d}{\alpha /\beta}\right)$, where *α*/*β* is the alpha/beta ratio of the tissue (assumed to be 10 Gy for NSCLC and 3 Gy for normal lung tissue), *n* is the total number of fractions of radiation therapy and *d* is the dose per fraction.

The BED_10_ is used to predict the response of tumor, and also of acutely-responding normal tissues (e.g. skin and mucosa). A higher BED_10_ is associated with a higher chance of local control, and a value of BED_10_ ≥ 100 is considered a minimum threshold for SABR [14]. The BED_3_ is used to predict the response of normal lung parenchyma (i.e. the risk of radiation pneumonitis). A lower BED_3_ is expected to be associated with a reduced risk of pneumonitis. However, efforts to reduce the BED_3_ will also lead to reductions in BED_10_, compromising the effectiveness of SABR. (For specific BED_10_ and BED_3_ values used in the studies included in the meta-analysis, see Additional file 1).

### Rationale for starting at a standard SABR dose

During the design of this trial in 2016–17, several options were considered and ultimately discussed at a meeting of the Canadian Pulmonary Radiotherapy Investigators’ (CAPRI) Group at the September 2017 Canadian Association of Radiation Oncology Annual Meeting. Two main alternative approaches were considered:

- **Option 1**: Conduct a phase II trial using a standard dose of SABR that has a high chance of local control; specifically, 50 Gy in 5 fractions (BED_10_ = 100), with careful attention to minimization of lung dose, and built-in dose de-escalation if toxicity is excessive.
- **Option 2.** Conduct a phase I trial starting with a less-potent dose of radiation to potentially minimize toxicity, and then escalate the dose thereafter. This would be expected to result in lower local control.

The CAPRI concluded that option 2 was unfavorable for 3 reasons: it would subject patients to a higher risk of local recurrence, which itself could be expected to be fatal; it is unclear that reducing to the lower dose levels would impact toxicity substantially; and many attendees stated that a common current approach outside of a trial is to offer full-dose radiotherapy after a careful discussion with patients.

There is precedent for this approach, as two other phase I trials of SABR have started at therapeutic doses: RTOG 0813 for central tumors (started at 50 Gy in 5 fractions before escalating to 60 Gy in 5 fractions) and SUNSET for ultra-central tumors (starting at 60 Gy in 8 fractions, currently underway) [15].

### Rationale for primary endpoint and study design

Important outcomes for patients with NSCLC and ILD include both toxicity and oncologic outcomes, and both were considered as possible primary endpoints.

Pulmonary toxicity was not chosen as a primary endpoint because it is very difficult to distinguish between radiation pneumonitis and ILD exacerbations. The patients included herein are expected to develop ILD exacerbations as part of the natural history of their ILD, and they are at risk of ILD-related death, which is difficult to distinguish from pneumonitis-related death. Erroneous attribution of ILD exacerbations to radiation could lead to the incorrect conclusion that the radiation dose should be lowered. In essence, the subjectivity of assigning the cause of toxicity should not be part of the primary outcome due to the risk of misattribution. However, pulmonary toxicity outcomes will be closely monitored in this trial.

Overall survival (OS) was chosen as the primary outcome as it objectively reflects the potential benefits of treatment (i.e. extended survival), the harms of treatment (grade 5 toxicity), and the natural history of the ILD disease process itself. Since the median survival of untreated Stage I NSCLC in inoperable patients is consistently reported as < 1 year (as noted above), we hypothesize that the use of SABR can achieve a median OS of > 1 year in patients with ILD. Though pulmonary toxicity is not to be the primary outcome in this trial, it will nevertheless be closely monitored to determine whether subsequently-enrolled patients will receive a deescalated radiation regimen.

SABR will be considered worthwhile if median OS is > 1 year with an acceptable risk of toxicity, defined as a grade 3 or 4 pulmonary toxicity rate of less than 35% and a risk of treatment-related death of less than 15%.

## Methods/design

This is a prospective phase II study of SABR in patients with co-existent interstitial lung disease, to determine oncologic and toxicity outcomes. Patients will be divided into 3 separate cohorts based on the ILD-GAP index (Table 1).

**Fig. 1:**
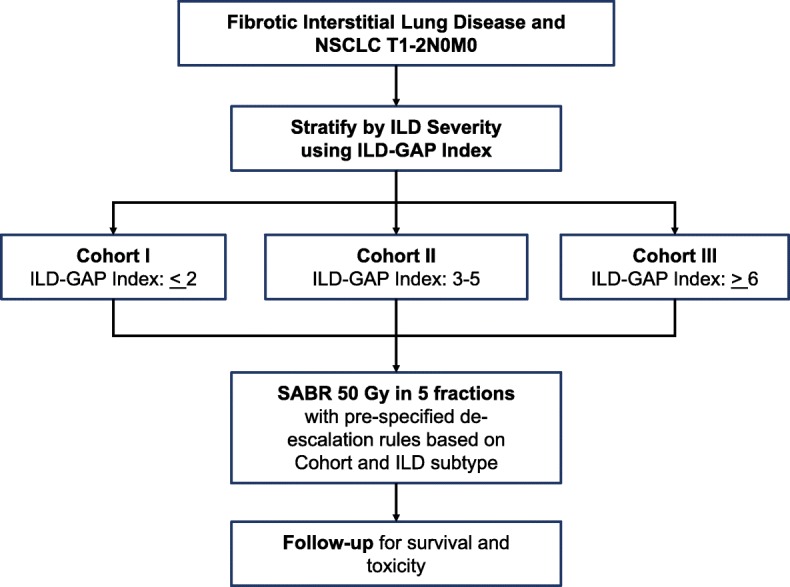
Study Schema. Patients with T1–2N0M0 non-small cell lung cancer and co-existing fibrotic interstitial lung disease will be enrolled in this single-arm study. Analysis will be stratified by interstitial lung disease severity. NSCLC: non-small cell lung cancer, ILD: interstitial lung disease, ILD-GAP: interstitial lung disease – gender/age/physiology model

The Study Schema can be found in Fig. 1. The required sample size is 39. Patients who meet eligibility criteria but decline to pursue radiation and patients for whom SABR cannot be delivered at the recommended doses due to inacceptable doses to normal tissues at risk will be asked to consent to limited ongoing follow-up only.

## Objectives

### Primary endpoint

- Overall survival: defined as time from enrollment to death from any cause

### Secondary endpoints

- CTC-AE 4.0 toxicity
  - Including risk of treatment-related mortality within 6 months of radiotherapy.
  - Including an analysis of pulmonary toxicity stratified by anti-fibrotic drug use
- Progression-free survival: defined as time from enrollment to death from any cause or any progression of disease (local, regional or distant)
- Local control
- Changes in cough severity (using a 10 cm visual analogue scale)
- Rates of acute-exacerbation of IPF (in IPF patients), defined using Collard 2016 criteria:
  - Acute worsening or development of dyspnea > 1 month duration
  - Computed tomography (CT) with new bilateral ground-glass opacity and/or consolidation superimposed on a background pattern consistent with usual interstitial pneumonia pattern
  - Deterioration not fully explained by cardiac failure or fluid overload
- Rates of acute-exacerbation of ILD (in non-IPF patients), defined using the same Collard 2016 criteria
- Quality of Life (Functional Assessment of Cancer Therapy – Lung (FACT-L) and EuroQol 5-Dimensional 5-Level (EQ-5D-5L))
- Changes in ILD severity as qualitatively measured on HRCT (i.e. HRCT fibrosis score and HRCT total disease score)
- Changes in pulmonary function tests, including diffusing capacity of the lungs for carbon monoxide (DLCO), forced expiratory volume in 1 s (FEV_1_), and forced vital capacity (FVC).
- Exploratory quantitative analysis of HRCT features (i.e. radiomics)
- Analysis of outcomes for patients who otherwise meet the study enrollment criteria but decline radiotherapy.

## Patient selection

### Inclusion criteria

- Stage T1–2, N0, M0 (American Joint Committee on Cancer (AJCC) Staging, 8th Edition – i.e. tumor size ≤5 cm) prior to registration.
- Not a candidate for surgical resection, determined by any of the following:
  - Consultation with a thoracic surgeon
  - Discussion at multidisciplinary tumour conference with a surgeon present
  - Patient refusal of surgery
- Pathologically (histologically or cytologically) proven diagnosis of non-small cell lung cancer (NSCLC) is not required, but strongly recommended.
  - If the risk of biopsy is unacceptable, pathologic confirmation is not required providing there is growth over time on CT imaging and/or fluorodeoxyglucose (FDG) avidity that is strongly suggestive of a primary NSCLC.
- Eastern Cooperative Oncology Group (ECOG) performance status 0–3.
- Age ≥ 18.
- Life expectancy > 6 months.
- Fibrotic interstitial lung disease of any subtype, as diagnosed by a respirologist/pulmonologist and confirmed by central review.

### Exclusion criteria

- Prior invasive malignancy except non-melanomatous skin cancer, unless the patient has been disease free for at least of 2 year.
- Prior thoracic radiotherapy
- Plans for the patient to receive other local therapy while on this study, except at disease progression;
- Plans for the patient to receive systemic therapy (standard chemotherapy, biologic targeted agents or immunotherapy) while on this study, except at disease progression. Patients are allowed to receive anti-fibrotic agents used in the treatment of IPF or non-IPF fibrotic ILD (e.g. nintedanib, pirfenidone), or steroids, if those are part of their current ILD treatment regimen. Other immunosuppressive drugs such as mycophenolate, azathioprine, cyclophosphamide, and rituximab must be stopped for 2 weeks prior and 2 weeks after treatment.
- Active pregnancy
- Concurrent administration of any drugs with known radiosensitive effects (e.g. Methotrexate).

## Pre-treatment evaluation

- History/physical examination within 4 weeks prior to registration, including documentation of current medications and home oxygen use.
- HRCT scan of the thorax (with contrast unless medically contraindicated) within 12 weeks of registration. Slice thickness must be 1.5 mm or less with continuous (i.e. non-gapped) images. Each centre must communicate with their radiologists to ensure that CT scans are done using protocols adequate for ILD. The primary tumor dimensions will be measured on CT.
- Positron emission tomography (PET) scan of the entire body within 8 weeks of registration, using FDG.
- Pre-bronchodilator spirometry, lung volumes and diffusion capacity within 8 weeks prior to treatment.
- Assessment by a respirologist/pulmonologist within 4 weeks of enrollment, for the following purposes:
  - To optimize pulmonary function and treatment of ILD, if possible.
  - To document subtype of ILD, and whether possible, probable or definite IPF exists.
  - To document key clinical features related to ILD (per a questionnaire), required for central review.
- Mediastinal lymph node sampling by any technique is **allowed** but **not required**. Hilar or mediastinal lymph nodes ≤1 cm in short-axis diameter with no pathological PET uptake will be considered N0. Patients with hilar or mediastinal lymph nodes > 1 cm in short-axis diameter on CT or lymph nodes with abnormal PET uptake may still be eligible if biopsies of suspicious lymph nodes are negative for malignancy.
- Assessment for brain metastases is not mandated unless suspicious symptoms are present, in which case a contrast-enhanced magnetic-resonance imaging (MRI) of the brain is required. If MRI is contra-indicated due to claustrophobia or metal implants (or other reason), then CT is allowed.

### Central review

A central review of the standardized clinical data, HRCT, and any previous lung biopsies (if available) will be done to confirm subtype of ILD. The central review team will include a pathologist, respirologist, and radiologist.

The following information will be required for central review:

- Clinical/Laboratory Data: Including clinical history, symptoms with date of onset, smoking history (including pack years and quit date), exposures, CTD features, serology (normal and abnormal), pulmonary function tests, and the subtype of ILD as determined by the treating respirologist.
- Pathology: slides (to be returned) and pathology reports
- Radiology: Digital Imaging and Communications in Medicine (DICOM) images of thoracic CT scans (through Quantitative Imaging for Personalized Cancer Medicine (QIPCM)) and radiology reports

Patients will be assigned to a category of fibrotic ILD (i.e. IPF, idiopathic non-specific interstitial pneumonia, CTD-ILD, hypersensitivity pneumonitis, Unclassifiable ILD/other) and an ILD-GAP score will be assigned.

## Radiation therapy

### Concurrent medications

During radiation, patients are allowed to receive anti-fibrotic agents (e.g. nintedanib) or steroids, if those are part of their current ILD treatment regimen. Other immunosuppressive drugs such as mycophenolate, azathioprine, cyclophosphamide, and rituximab must be stopped for 2 weeks prior and 2 weeks after treatment.

### Dose/fractionation

The total dose will be 50 Gy in 5 fractions, given every second day. In the event that the dose is de-escalated to 50 Gy in 10 fractions or 45 Gy in 15 fractions, then the same prescribing principles apply (i.e. using a SABR-like technique) but these latter two fractionations are given daily.

### Immobilization

A stable and consistent immobilization technique is advised for all patients and all fractions to maximize inter-fraction reproducibility. Patients should be comfortably immobilized during treatment to minimize unnecessary motion due to discomfort and maximize intra-fraction reproducibility. Different immobilization systems may be utilized including a thermoplastic shell, alpha-cradle, Vac-Lok bag or none, per institutional standard. The immobilization setup should be identical to that used at simulation time.

### Imaging

Patients will undergo planning CT simulation with a 4-dimensional (4D)-CT, unless being treated with a system that accounts for motion without 4D-CT (e.g. CyberKnife). The use of IV contrast is not required. Axial CT images will be obtained throughout the region of interest**.** At each center, the local physicist or CT-simulation therapists will review the 4D-CT images and will perform the following quality assurance procedures: ensuring all end inspiration (0%) tags exist and are in the right place; ensuring that the quality of the 4D-CT images are acceptable (determined by Physics or CT-simulation therapists if standard at that institution); and that motion measurements in all 3 directions are performed.

### Motion management

Motion assessment is mandatory. The specific target motion of each patient must be quantified to determine if additional motion management strategies are required. If the tumor excursion is ≤1 cm, the internal target volume (ITV) approach is sufficient for treatment planning and dose-volume histogram (DVH) analysis, although standard institutional motion management strategies may be used as well. The design of the target volume should cover the primary lung cancer during breathing. If the tumor excursion is > 1 cm, acceptable motion management techniques for reducing target motion ≤1 cm include abdominal compression, active breathing control, active breath hold, free-breathing gating, gated breath hold or robotic tumor tracking (direct soft tissue or fiducial tracking).

### Volume definitions

The target lesion will be outlined by an appropriately trained physician and designated the gross tumor volume (GTV) on both the inspiratory and expiratory CT images. The target will generally be drawn using CT pulmonary windows; however, soft tissue windows should be used to avoid inclusion of adjacent vessels, atelectasis, mediastinal or chest wall structures within the GTV. This target will not be enlarged whatsoever (i.e. no “margin” for presumed microscopic extension); rather, it will only include areas consistent with gross tumor (i.e., the GTV and the Clinical Target Volume, CTV, are identical). PET images may be used at the discretion of the treating oncologist, but consistent with International Atomic Energy Agency (IAEA) guidelines, CT should be used to determine the boundary between tumor and normal tissue, as PET images are subject to thresholding effects.

An ITV is generated from the outlined GTV on the inspiratory and expiratory CT datasets. An additional 0.5 cm will be added in all planes to constitute the planning treatment volume (PTV). Depending on institutional practice, the Exhale phase may be used as a primary dataset for treatment planning with fusion of the inhale, maximum intensity projection (MIP) and average. Alternatively, the Average phase may also be used as the primary dataset. The GTVs contoured on these datasets will define the internal GTV (IGTV). This contour may be used to define the PTV using standard margins or for image registration with cone beam CT (CBCT).

For respiratory gating treatment, if used, planning will be performed on a subset average CT dataset (usually labeled either 30–60% Avg CT or 40–70% Avg CT) generated by Physics. This is an average CT over the intended gated interval. Therefore, the GTV that is delineated on this scan will incorporate residual motion in the intended gated interval. The 0% phase will also be fused to this dataset. The PTV for planning will include the GTV delineated on the subset average CT and setup uncertainty. The GTV_0% should also be delineated and combined with the GTV delineated on the subset average CT to define an additional volume labeled IGTV_CBCT. This contour may be used for image guidance with CBCT only.

### Dosimetry

Treatment can be delivered using static beams (either 3D-conformal radiotherapy or intensity-modulated) or rotational therapy (volumetric modulated arc therapy, or Tomotherapy). Only photon (X-ray) beams of 10 MV or less produced by linear accelerators will be used.

For purposes of dose planning and calculation of monitor units for actual treatment, this protocol will require tissue density heterogeneity correction. Successful treatment planning will require accomplishment of all of the following criteria:

- Prescription isodose surface: The prescription isodose surface will conformally cover 95% of the target volume (PTV), such that 99% of the PTV will also receive at least 90% of the prescription dose. (PTV D95% = 100% of prescription dose and D99% = 90% of prescription dose)
- Target dose heterogeneity: The dose at the prescription isodose surface must be between 60 and 90% (inclusive) of the dose at the normalization point of the plan. The prescription isodose surface is typically 80% of the dose at the normalization point, though the allowed range enables flexibility to optimize dose to organs at risk (OARs), especially the lung.
- High dose spillage (as per the RTOG 0813 [16] protocol):
  - Location: Areas with dose > 105% of the prescription dose should be located primarily within the PTV itself. The cumulative volume outside the PTV volume with dose > 105% of prescription dose should be no more than 15% of the PTV volume.
  - Volume: Conformality of PTV coverage will be determined by the R100 (the ratio of the volume of the prescription isodose to the volume of the PTV), R50 (The ratio of the volume of 50% of the prescription dose isodose to the volume of the PTV), and D2cm (the maximum total dose over all fractions in Gray (Gy) to any point 2 cm or greater away from the PTV in any direction), as per Table 3.
- All critical organ dose-volume constraints in Table 2 must be respected. **Lung dose constraints may not be exceeded for any reason.**

**Table 2:** Recommended dose constraints for non-lung organs. 5-fraction and 8-fraction constraints based on RTOG 0813 protocol [16], SABR-COMET protocol [17], and Timmerman et al [18]. Constraints for other fractionation schemes are derived using BED_3_

Structure		Number of Fractions		
		5	10	15
Spinal Cord (contoured as spinal canal)	Max	30 Gy	34.5 Gy	39.5 Gy
	< 0.25 cc	22.5 Gy	29 Gy	33 Gy
	< 1.2 cc	13.5 Gy	17 Gy	19 Gy
Esophagus	Max	35 Gy	43.5 Gy	50.5 Gy
	< 5 cc	27.5 Gy	35.5 Gy	41 Gy
Ipsilateral Brachial Plexus	Max	32 Gy	42.5 Gy	49 Gy
	< 3 cc	30 Gy	39.5 Gy	45.5 Gy
Heart	Max	38 Gy	50 Gy	58.5 Gy
	15 cc	32 Gy	42.5 Gy	49 Gy
Great Vessels	Max	53 Gy	71.5 Gy^a^	84 Gy^a^
	10 cc	47 Gy	63.5 Gy	74.5 Gy^a^
Trachea and Ipsilateral Bronchus	Max	38 Gy	43.5 Gy	50.5 Gy
	4 cc	18 Gy	23 Gy	26 Gy
Skin	Max	32 Gy	42.5 Gy	49 Gy
	10 cc	30 Gy	39.5 Gy	45.5 Gy
Stomach	Max	32 Gy	43.5 Gy	50.5 Gy
	10 cc	28 Gy	37 Gy	42.5 Gy
Liver	At least 700 cc	< 21 Gy	< 23.5 Gy	< 26.5 Gy
Chest Wall	Keep as low as reasonably achievable, do not compromise PTV, respect PTV conformality parameters			

^a^Although calculated tolerance dose is much higher, seek to limit any point of the organ to 110% of prescription dose. Non-adjacent wall must always be limited to 105% (see below)

### Critical structures

Dose-volume histograms must be generated for each critical structure so that the dose-volume constraints can be evaluated. Instructions for contouring the critical structures are provided below and reflect common radiotherapy contouring procedures, as per RTOG 0813 [16]:

- Spinal cord: the spinal cord is contoured based on the bony limits of the spinal canal. The spinal cord should be contoured on every slice starting at least 10 cm superior to the most superior extent PTV to at least 10 cm inferior to the most inferior extent of the PTV.
- Esophagus: the esophagus will be contoured using the mediastinal window on CT corresponding to the mucosal, submucosa, and all muscular layers of the esophagus. The esophagus should be contoured on every slice starting at least 10 cm superior to the most superior extent of the PTV to at least 10 cm inferior to the most inferior extent of the PTV.
- Ipsilateral brachial plexus: the ipsilateral brachial plexus originates from the spinal nerves exiting the neuroforamina from C5 to T1. The detailed contouring procedures can be found in the RTOG contouring atlas [19].
- Heart: the heart and the pericardial sac will be contoured together as the heart critical structure. The superior aspect (or base) of the heart will begin at the level of the inferior aspect of the aortic arch and extend inferiorly to the apex of the heart.
- Trachea and proximal bronchial tree: the trachea and proximal bronchial tree will be contoured as two separate structures using mediastinal windows on CT to correspond to the mucosal, submucosa and cartilage rings and airway channels associated with these structures. For this purpose, the trachea will be divided into two sections: the proximal trachea and the distal 2 cm of trachea. The proximal trachea will be contoured as one structure, and the distal 2 cm of trachea will be included in the structure identified as proximal bronchial tree.
  - Contouring of the proximal trachea should begin at least 10 cm superior to the extent of the PTV or 5 cm superior to the carina (whichever is more superior) and continue inferiorly to the superior aspect of the proximal bronchial tree.
  - The proximal bronchial tree will include the most inferior 2 cm of distal trachea and the proximal airways on both sides. The following airways will be included according to standard anatomic relationships: the distal 2 cm of trachea, the carina, the right and left mainstem bronchi, the right and left upper lobe bronchi, the intermedius bronchus, the right middle lobe bronchus, the lingular bronchus, and the right and left lower lobe bronchi. Contouring of the lobar bronchi will end immediately at the site of a segmental bifurcation. If there are parts of the proximal bronchial tree that are within GTV, they should be contoured separately, as “proximal bronchial tree GTV”, not as part of the “proximal bronchial tree”.
- Whole lung: both the right and left lungs should be contoured as one structure. Contouring should be carried out using pulmonary windows. All inflated and collapsed lung should be contoured.
- PTV plus 2 cm: A quality-assurance structure 2 cm larger in all directions from the PTV is required to determine the maximum dose at any point 2 cm from the PTV. Most treatment planning systems have automatic contouring features that will generate this structure.
- Great vessels: the great vessels (aorta, venae cavae, pulmonary artery and pulmonary vein) will be contoured as a single structure using mediastinal windows to correspond to the vascular wall and all muscular layers of these great vessels. The great vessels should be contoured on every slice starting at least 5 cm superior to the most superior extent of the PTV to at least 5 cm inferior to the most inferior extent of the PTV. The distal extent of the right and left pulmonary arteries will be the bifurcation to the basal segmental arteries. The pulmonary veins will be contoured until the superior and inferior pulmonary veins bifurcate to the segmental and basal veins.
- Chest wall: the chest wall includes the ribs and intercostal muscles and nerves. It should be defined as a 2 cm anterior, lateral, and posterior, expansion of the lung, not including the lung itself, the mediastinum, diaphragm, GI structures, or the spinal cord.

### Dose constraints for organs besides lung parenchyma

Table 2 lists maximum dose limits to a point or volume for several critical organs, depending on the number of fractions. Note that the number of fractions will be 5 unless the dose is de-escalated due to safety reasons. These dose constraints have been calculated using the BED formula and are generally considered iso-toxic, meaning that they are considered to portend an equal risk of toxicity to those organs, across the different fractionation schemes. This is in distinction to the lung dose constraints below, which are NOT iso-toxic, meaning that the risk of toxicity increases with higher dose levels.

#### Note on central tumors

When the GTV tumor is within 2 cm of a central structure (e.g. heart, trachea), it will be considered a central tumor. In such a scenario, the dose constraints to central structures in Table 2 are often not achievable. These should be planned using the same approach as in the RTOG 0813 study, which determined the safe dose level of central tumors for a 5-fraction SABR regimen. In such an approach, the PTV coverage is not compromised, but the OAR dose is to be kept as low as possible. This applies to the esophagus, heart, great vessels, trachea, and ipsilateral bronchus. For the esophagus, great vessels, trachea, and ipsilateral bronchi, the non-adjacent wall of the structure limited to a maximum dose of 105% of prescription dose. In contrast, maximum doses from the above table for the spinal cord, brachial plexus, skin, and stomach are NOT to be exceeded.

### Constraints for lung parenchyma

The dose constraints to the lung are not iso-toxic, meaning that the risk of toxicity is highest with highest dose levels. The constraints below based are those used for 5-fraction SABR regimens in patients without ILD, meaning that with a higher number of fractions (e.g. 10, or 15), the risk of toxicity will decrease. The pulmonary constraints consist of 2 critical volume constraints, along with the R100, R50, D2cm and V20 (volume of lung receiving at least 20 Gy), all defined below.

Critical volume constraint for both lungs (right lung + left lung): a total critical volume of 1500 mL receives no more than 12.5 Gy. This value is calculated from the dose-volume histogram as the absolute volume of lung tissue receiving < 12.5 Gy. That volume must be at least 1500 mL.

The conformality and V20 constraints for this study will be those used for the RTOG 0813 study, reproduced in Table 3.

**Table 3:** Conformality and V20 constraints, as per the RTOG 0813 protocol [16]

PTV Volume (cc)	Ratio of prescription isodose volume to the PTV bolume (R100)		Ratio of 50% prescription isodose volume to the PTV volume, (R50)		Maximum dose (in % of dose prescribed) @ 2 cm from PTV in any direction, (D2cm) (Gy)		Percent of Lung receiving 20 Gy total or more (V20)	
	Deviation		Deviation		Deviation		Deviation	
	None	Minor	None	Minor	None	Minor	None	Minor
1.8	< 1.2	< 1.5	< 5.9	< 7.5	< 50.0	< 57.0	< 10	< 15
3.8	< 1.2	< 1.5	< 5.5	< 6.5	< 50.0	< 57.0	< 10	< 15
7.4	< 1.2	< 1.5	< 5.1	< 6.0	< 50.0	< 58.0	< 10	< 15
13.2	< 1.2	< 1.5	< 4.7	< 5.8	< 50.0	< 58.0	< 10	< 15
22.0	< 1.2	< 1.5	< 4.5	< 5.5	< 54.0	< 63.0	< 10	< 15
34.0	< 1.2	< 1.5	< 4.3	< 5.3	< 58.0	< 68.0	< 10	< 15
50.0	< 1.2	< 1.5	< 4.0	< 5.0	< 62.0	< 77.0	< 10	< 15
70.0	< 1.2	< 1.5	< 3.5	< 4.8	< 66.0	< 86.0	< 10	< 15
95.0	< 1.2	< 1.5	< 3.3	< 4.4	< 70.0	< 89.0	< 10	< 15
126.0	< 1.2	< 1.5	< 3.1	< 4.0	< 73.0	> 91.0	< 10	< 15
163.0	< 1.2	< 1.5	< 2.9	< 3.7	< 77.0	> 94.0	< 10	< 15

### Procedure if lung constraints cannot be met

**The lung constraints are critically important for this study.** If one or more of the lung constraints cannot be met, even after varying the prescription isodose surface percentage between 60 and 90%, PTV coverage must then be compromised. Target coverage may be reduced until 95% of the IGTV is receiving the 95% of the prescription dose, and 90% of the IGTV is receiving 99% of the prescription dose, with the PTV covered as well as possible while respecting lung constraints.

**If the lung dose constraints still cannot be met, the patient should be treated with a 10 or 15-fraction regimen.** Such patients treated with protracted regimens due to inability to meet dose constraints will not count toward the primary analysis, but should still be followed for toxicity and outcomes. Such patients will be reported as a separate cohort.

### Quality assurance

In order to ensure patient safety and effective treatment delivery, a robust quality assurance protocol is incorporated. The following requirements must be completed for each patient:

- Prior to treatment, each patient’s contours and radiation plan must be peer-reviewed at the local centre. This can include review by one other radiation oncologist, or discussion at quality assurance (QA) rounds.
- OAR dose constraints may be only exceeded with approval of the PI. Prior to plan approval, the dose to each organ at risk must be verified by the physicist or treating physician.
- All dose delivery for intensity-modulated plans (including arc-based treatments) will be confirmed before treatment by physics staff.
- Online imaging (Cone-beam CT, megavoltage CT or orthogonal KV with tumor tracking) will be used to verify patient positioning for each treatment. Ideally, direct tumour localization should be performed. For gated SABR treatments, direct tumour localization will be performed by matching the tumour position with the region of interest (ROI) defined by IGTV_CBCT. This will be followed by a gated 2-dimensional (2D)-kV in the anterior-posterior plane to verify the gating window. In the absence of direct tumour localization, reliable soft tissue surrogates are recommended. For non-gated radiotherapy, it is expected to match the image of the tumor with the ROI defined by the ITV.

### Quality Assurance for Centres Joining Study

Prior to opening the study, each participating research centre will be required to send to one of the Principal Investigators a mock treatment plan, to ensure that the treatment plans are designed in compliance with the protocol. The principal investigators will provide pertinent CT datasets. Centers that have previously been accredited for other trials (e.g. NRG Oncology, CAPRI, LUSTRE [20] or SABR-COMET [17]) must only send documentation of that accreditation.

## Adverse events

### Definitions

*Adverse Event (AE)* or reaction, as per definitions by the Common Terminology Criteria for Adverse Events [21], is any unfavorable and unintended sign (including an abnormal laboratory finding), symptom, or disease temporally associated with the use of a medical treatment or procedure that may or may *not* be considered related to the medical treatment or procedure.

*Serious Adverse Event (SAE)* or reaction as defined in the International Council for Harmonisation (ICH) Guideline: Clinical Safety Data Management: Definitions and Standards for Expedited Reporting, Section 2B [22] includes any untoward medical occurrence at any dose that:

- Results in death
- Is life-threatening (refers to an event in which the patient was at risk of death at the time of the event; it does not refer to an event which hypothetically might have caused death if it were more severe.)
- Results in persistent or significant disability/incapacity
- Requires in-patient hospitalization or prolongation of existing hospitalization
- Is a congenital anomaly/birth defect

Important medical events that may not be immediately life-threatening or result in death or hospitalization may be considered a serious adverse event, when, based upon medical and scientific judgment, they may jeopardize the patient or may require intervention to prevent one of the other outcomes listed in the definition above.

*Unexpected adverse event* or reaction is one that the nature and severity is not consistent with the applicable product information (e.g., Investigator’s Brochure or Product Monograph, described in the research ethics board (REB)/institutional review board (IRB) approved research protocol or informed consent document), or occurs with more than expected frequency.

### Radiation therapy adverse events

RT delivered in this protocol can adversely affect organs at risk, most notably lungs, airway, esophagus, pulmonary vessels, and heart/pericardium, as these organs may be in close proximity to the intended target (PTV) of RT.

- Cardiac and pericardial injury: though cardiac/pericardial toxicity is rare following conventionally-fractionated RT, such toxicity may be seen with SABR due to larger fraction sizes.
- Gastrointestinal/esophageal injury: SBRT can result in acute esophagitis and/or late esophageal stenosis or ulceration, with esophageal perforation occurring in the extreme cases.
- Central airway/bronchial injury: this injury may result in focal atelectasis and impair overall pulmonary status. Atelectasis can render assessment of tumor response very difficult. Investigators are referred to the strict criteria for progressive disease in the Disease Response and Progression section of this protocol to avoid such mis-characterization.
- Lung injury: radiation pneumonitis is a subacute inflammation of the end bronchioles and alveoli due to radiation exposure. Radiation fibrosis is a late manifestation of radiation injury to the irradiated lung. Radiation-induced lung injury has not been traditionally been a dose-limiting factor for SABR in healthy lung, though the risk seems to be significantly increased in patients with co-existing fibrotic ILD. Radiation pneumonitis can also be confused with other causes of respiratory deterioration including pneumonia, acute exacerbation of COPD, acute exacerbation of ILD and tumor recurrence. It is crucial for a Radiation Oncologist participating in this study to be aware of the potential differential diagnosis, as the symptomatology of radiation pneumonitis can mimic these other causes with fatigue, fever, shortness of breath, nonproductive cough, and a pulmonary infiltrate on chest radiography. For radiation pneumonitis, the infiltrate on chest radiography should include the area treated to a high dose of radiation but may extend outside of these regions. The infiltrates may be characteristically “geometric” though with modern radiotherapy techniques the region is likely to be ill-defined.

### Causality of pre-specified toxicity endpoints

Any Grade 3–5 toxicity listed in Table 4 will **automatically** be considered to be possibly, probably or definitely related to treatment, unless there is clear evidence that the adverse event is unrelated or unlikely to be related. The latter instance may occur, for example, in the case of a cardiac event in a patient with an upper lobe tumor and negligible heart dose, or esophageal issues in a patient who received negligible esophageal dose. The final decision will be made by the members of the data safety monitoring committee (DSMC). See below for causality definitions.

**Table 4:** Pre-specified adverse events

Structure	Adverse Event
Cardiac and Pericardial	• Acute coronary syndrome
	• Aortic valve disease
	• Atrial fibrillation
	• Atrial flutter
	• Atrioventricular block
	• Conduction disorder
	• Constrictive pericarditis
	• Heart failure
	• Left ventricular systolic dysfunction
	• Myocarditis
	• Pericardial effusion
	• Pericardial tamponade
	• Pericarditis
	• Cardiomyopathy
	• Cardiac disorders-others
Gastrointestinal	• Dyspepsia
	• Dysphagia
	• Esophageal fistula
	• Esophageal hemorrhage
	• Esophageal necrosis
	• Esophageal obstruction
	• Esophageal stenosis
	• Esophageal perforation
	• Esophagitis
Pulmonary/mediastinal	• Atelectasis
	• Bronchial fistula
	• Bronchial obstruction
	• Bronchopleural fistula
	• Bronchopulmonary hemorrhage
	• Dyspnea
	• ILD or IPF exacerbation
	• Pneumonitis
	• Tracheal/Pulmonary fistula
	• Tracheal stenosis
	• Mediastinal hemorrhage
	• Pulmonary disorders-others

### Causality definitions

An adverse event or reaction is considered **related** to the research intervention (i.e. radiotherapy) if there is a reasonable possibility that the reaction or event may have been caused by the research intervention (i.e. a causal relationship between the reaction and the research intervention cannot be ruled out by the investigator(s)).

The relationship of an AE to the study treatment (causality) will be described using the following definitions:

- Unrelated: Any adverse event for which there is evidence that an alternative etiology exists or for which no timely relationship exists to the administration of the study treatment and the adverse event does not follow any previously documented pattern. The adverse event, after careful consideration by the investigator, is clearly and incontrovertibly due to causes other than the intervention.
- Unlikely: Any adverse event for which the time relationship between the study treatment and the event suggests that a causal relationship is unlikely and/or the event is more likely due to the subject’s clinical condition or other therapies concomitantly administered to the subject.
- Possible: Any adverse event occurring in a timely manner after the administration of the study treatment that follows a known pattern to the intervention and for which no other explanation is known. The adverse event, after careful consideration by the investigator, is considered to be unlikely related but cannot be ruled out with certainty.
- Probable: Any adverse event occurring in a timely manner after the administration of the study treatment that follows a known pattern to the intervention and for which no other explanation is known. The adverse event, after careful consideration by the investigator, is believed with a high degree of certainty to be related to the intervention.
- Definitely Related: Any adverse event occurring within a timely manner after administration of the study treatment that is a known sequela of the intervention and follows a previously documented pattern but for which no other explanation is known. The adverse event is believed by the investigator to be incontrovertibly related to the intervention.

### Severity

The severity of adverse events will be evaluated using the Common Terminology Criteria for Adverse Events (CTCAE) v4.0 grading scale [21].

- Grade 1: Mild
- Grade 2: Moderate
- Grade 3: Severe
- Grade 4: Life-threatening or disabling
- Grade 5: Death

Note: The term “severe” is a measure of intensity: thus a severe adverse event is not necessarily **serious**. For example, nausea of several hours’ duration may be rated as severe, but may not be clinically serious.

### Immediately reportable adverse events

Any grade 4 or 5 adverse reaction that is definitely, probably, or possibly the result of experimental treatment (i.e. radiotherapy) must be verbally reported to the Principal Investigator and Coordinating Centre within 24 h of discovery, and to the approving REB if necessary as per their reporting guidelines.

Local and non-local SAEs will be reported to the applicable REB as per their reporting guidelines. All **serious**, **unexpected** adverse events or reactions **regardless of causality** will be reported within **7** days of discovery to the pertinent REB, or as required as per the REB guidelines.

Note: conditions that are NOT related to protocol treatment or baseline Interstitial Lung Disease are not considered a SAE in this protocol.

## Subject withdrawal

Subjects may voluntarily discontinue participation in the study at any time. If a subject is removed from the study, the clinical and laboratory evaluations that would have been performed at the end of the study should be obtained. If a subject is removed because of an adverse event, they should remain under medical observation as long as deemed appropriate by the treating physician.

## Followup and assessment of efficacy

Patients will be seen in follow-up as per Table 5. At each visit, a history and physical examination will be conducted by the oncologist, and CTCAE toxicities recorded, and quality of life questionnaires will be completed.

**Table 5:** Follow-up evaluations

	Before Treatment	Year 1^a^	Year 2–5^a^
History and Physical	X	3, 6, 9, 12 mo	18 mo, 24 mo, then annually
Assessment by Respirology/Pulmonology	X	At least every 6 months	At least annually
Central review of ILD diagnosis	X		
PET/CT	X		
Brain MRI	If symptoms (see Pre-treatment Evaluation)		
Mediastinal staging	Optional (see Pre-treatment Evaluation)		
HRCT chest	X	3, 6, 12 mo	18 mo, 24 mo, then annually
PFTs^b^	X	6, 12 mo	18 mo, 24 mo, then annually
Toxicity Scoring	X	3, 6, 9, 12 mo	18 mo, 24 mo, then annually
FACT-L, EQ-5D-5L QOL scoring and cough severity scale	X	3, 6, 9, 12 mo	18 mo, 24 mo, then annually

^a^All times are measured from end of radiation

^b^To avoid duplication of PFTs, if the patient is having PFTs done by other physicians (e.g. respirologists), the PFTs must only be +/− 2 months of the dates stated above to be acceptable

CT chest will be repeated at 3, 6, 12, 18 and 24 months, then annually. Additional imaging or laboratory investigations may be carried out at the discretion of the oncologist. Pulmonary function tests will be repeated at 6, 12, 18, and 24 months.

### Follow-up of patients declining study intervention

It is expected that some of patients who are offered participation in this trial decline the intervention, and thus would normally decline to enter the study. Patients who decline enrollment on the trial because they do not want to receive radiation will be asked to consent to follow-up phone calls (at 3-monthly intervals for 2 years) and prospective inclusion of their clinical data collected from the medical record as part of a cohort of patients declining radiotherapy. The phone calls will ask the participant about pulmonary symptoms (in the same manner as toxicity scoring is done in patients who receive radiotherapy), and any other cancer treatments received.

### Measurement of response

Survival outcomes: OS will be measured as time until death from any cause, and progression-free survival as time to either local, regional or distant progression or death, whichever occurs first.

### Disease response and progression

Assessment of response on imaging after SABR is difficult, as fibrotic changes in the lung may obscure tumor measurements. Nonetheless, the Response Evaluation Criteria in Solid Tumors (RECIST) 1.1 [23] will be used.

Response to SBRT will be assessed by a central review process, where the PI and at least one other member of the trial committee will review the diagnostic imaging prior to data analysis to determine response. Histological confirmation and/or PET scan may be recommended to assess cases suspicious for recurrence. Any lesions that exhibit growth as per RECIST 1.1 criteria will be counted as progression, unless subsequent scans show a period of no growth of > 6 months.

## Statistical considerations

### Statistical design and sample size

The primary endpoint of this study is overall survival in patients able to be treated with a dose of 50 Gy in 5 fractions, with a comparison to historical controls of untreated medically inoperable patients stage I NSCLC, which consistently have survivals of less than 1 year, as described in the Background section. Demonstration in this study that median OS after SABR is statistically greater than a historical control of < 12 months would indicate that SABR is worthwhile in this population of ILD patients, if toxicity is within acceptable limits.

A sample size of 39 patients provides >80% power to detect an OS improvement of >20% at 1 year, compared to a historical control of <50% (i.e. 70% vs. 49%), using a one-sample, one sided binomial test at the 0.05 significance level, assuming 10% dropout or loss to follow-up before one year. All cohorts will be combined for this primary analysis.

SABR will be considered worthwhile if OS is > 1 year, the risk of grade 3 or 4 pulmonary toxicity is < 35%, and the risk of treatment-related mortality is < 15%.

### Data safety monitoring

The DSMC will meet every 6 months after study initiation to review toxicity outcomes, but will also be notified in-between meetings if more than 2 grade 5 treatment-related toxicities occur within the first 7 patients enrolled to any cohort, or for any subtype of ILD regardless of cohort.

The dose will be de-escalated if 2 or more of the first 7 patients in a specific cohort or with a specific ILD subtype experience grade 5 treatment-related toxicity within 6 months of treatment. For example, if 2 of the first 7 patients in cohort III experience grade 5 toxicity within 6 months of treatment, then the dose will be de-escalated for all future patients in cohort III. Similarly, if 2 of the first 7 patients with CTD-ILD experience grade 5 toxicity within 6 months of treatment, then the dose will be de-escalated for all future patients with CTD-ILD.

After each de-escalation, if 2 of the next 7 patients in that cohort develop grade 5 toxicity within 6 months of treatment, then de-escalation will be repeated. The dose will be de-escalated to 50 Gy in 10 fractions, then 45 Gy in 15 fractions, using the same dose prescription techniques as described above. After 45 Gy in 15 fractions, if further de-escalation is needed then that cohort will be closed.

Using binomial distribution modelling, the performance characteristics of this approach are displayed in Table 6.

**Table 6:** Probability of dose de-escalation based on true risk of death in a given patient cohort

True Risk of Death in a Given Cohort	Probability of De-Escalation for that Cohort
0.08	10%
0.15	28%
0.30	67%

^a^At risk levels higher than 30%, which are thought to be extremely unlikely, dose de-escalation probabilities rise further, and chances of de-escalation within the first few patients are very high

If de-escalation occurs for a certain cohort, then further patients enrolled in that cohort will be treated with de-escalated doses but will not count towards the overall accrual goal (i.e. there will be 39 patients treated with 50 Gy in 5 fractions, although ALL patients treated will be followed for outcomes and reported). Final results will be stratified by dose level: they will be presented for all patients, patients treated with the target dose of 50 Gy in 5 fractions, and for patients treated at de-escalated doses.

## Ethical considerations

### Institutional review board (IRB) / research ethics board (REB)

The protocol (and any amendments), the informed consent form, and any other written information to be given to subjects has been reviewed and approved by the Ontario Cancer Research Ethics Board, operating in accordance with the current federal regulations. The Clinical Trials Ontario Project ID is 1488. Any institution opening this study will obtain local IRB/REB approval prior to local initiation.

### Informed consent

The written informed consent form is to be provided to potential study subjects and should be approved by the local IRB/REB and adhere to principles of the International Council for Harmonisation Guidelines for Good Clinical Practice (ICH GCP), which have their origins in the Declaration of Helsinki. The study consent form can be found in Additional file 2. The investigator is responsible for obtaining written informed consent from each subject, or if the subject is unable to provide informed consent, the subject’s legally acceptable representative, prior to beginning any study procedures and treatment(s). The investigator should inform the subject, or the subject’s legally acceptable representative, of all aspects of the study, including the potential risks and benefits involved. The subject should be given ample time and opportunity to ask questions prior to deciding about participating in the study and be informed that participation in the study is voluntary and that they are completely free to refuse to enter the study or to withdraw from it at any time, for any reason. The informed consent must be signed and dated by the subject, or the subject’s legally acceptable representative, and by the person who conducted the informed consent discussion. A copy of the signed and dated written informed consent form should be given to the subject or the subject’s legally acceptable representative. The process of obtaining informed consent should be documented in the patient source documents.

### Confidentiality of subject records

The names and personal information of study participants will be held in strict confidence. All study records (case report forms, safety reports, correspondence, etc.) will only identify the subject by initials, month and year of birth, and the assigned study identification number. The investigator will maintain a confidential subject identification list (Master List) during the course of the study. Access to confidential information (i.e., source documents and patient records) is only permitted for direct subject management and for those involved in monitoring the conduct of the study (i.e., Sponsors, contract research organizations, representatives of the IRB/REB, and regulatory agencies). The subject’s name will not be used in any public report of the study.

## Optional sub-study (London site only): aspire-MRI

The ASPIRE-MRI sub-study will consist of separate pulmonary ventilation and perfusion scanning done pre- and post-radiotherapy using hyperpolarized ^3^He and/or ^129^Xe magnetic resonance imaging at the Robarts Research Institute. This is outlined in Additional file 3. The consent for this sub-study is in Additional file 4.

## Authorship

Upon completion of this project, the results will be published in a peer-reviewed journal and presented at conferences. As noted above, each cohort may be reported separately.

Final decisions on authorship will be made by the trial steering committee, and will be commensurate with the relative accrual of each center and the amount of individual contribution, including study design, patient accrual, and data analysis. Authorship on all publications must follow the International Committee of Medical Journal Editors (ICMJE) guidelines (www.icjme.org). In general, the principal investigator will be expected to lead all publications and presentations of primary endpoint data as first author. The authorship group otherwise consists of a representative from each centre accruing patients, along with the trial steering committee, provided that co-authors participate in the authorship process as outlined by ICMJE. Separate substudies or reports of secondary endpoints may be led by other investigators within the group.

## Central imaging collection

Anonymized imaging data will be collected through the Quantitative Imaging for Personalized Cancer Medicine (QIPCM) platform (qipcm.technainstitute.com). QIPCM provides centralized storage and data analysis tools for medical imaging, and is compliant with national and international privacy regulations. A flowchart of enrollment and central review can be found in Additional file 5.

Scans collected by QIPCM will be: baseline CT scan done at the time of enrollment, radiation planning scan, and radiation dose distribution, and follow-up CTs. The protocol for uploading of images from remote sites will be provided in a separate Imaging Collection Handbook.

## Discussion

ASPIRE-ILD is a Phase II trial that aims to assess outcomes after SABR in patients with medically-inoperable ES-NSCLC and co-existing fibrotic ILD. As a growing number of patients undergo SABR for lung lesions with diagnosed and undiagnosed ILD, it is increasingly important to evaluate the outcomes of SABR in this population of patients. A summary of the trial information in the format of the World Health Organization Trial Registration Data Set can be found in Additional file 6. This trial protocol has undergone peer review by the Canadian Cancer Clinical Trials Network.

The optimal dose of SABR is unclear in this setting. In the event that a significant level of treatment-related mortality is observed, the SABR dose can be de-escalated from 50 Gy in 5 fractions (BED_10_ = 100 Gy_10_) to 50 Gy in 10 fractions (BED_10_ = 75 Gy_10_) then 45 Gy in 15 fractions (BED_10_ = 58.5 Gy_10_). Forty-five Gy in 15 fractions is a hypofractionated dose regimen that is not usually considered “stereotactic”. However, in the interest of determining whether a safe radiation dose and fractionation exists at all for patients with fibrotic ILD, 45 Gy in 15 fractions was included as a minimum dose level that could have a possibility of obtaining lasting local control. Additionally, the prescription method for 45 Gy in 15 fractions in this trial will remain “stereotactic”, with dose being prescribed mainly to the 80% isodose line with high conformity and image guidance standards. This would mean that the central dose maximum could still reach > 56 Gy in 15 fractions (BED_10_ = 77 Gy_10_) if the minimum dose level was used.

A secondary study (Additional file 3) is also proposed for the London site to investigate the role of advanced imaging techniques in visualizing the effect of SABR on the structure and function of the lung. Xenon-129 MRI will be performed for consenting patients to investigate the change in ventilation defect after SABR. Visualizing functional changes in lung parenchyma after SABR will be highly valuable in the continued study of radiation-induced lung injury. In addition to the main study focusing on the outcomes of SABR, patients who decline SABR will also be asked for consent to enroll in an observation-only group of the study to better understand the natural history and prognosis of co-existing ES-NSCLC and ILD.

Limitations of this study include potentially shorter available follow-up times due to the usually limited prognosis from comorbid ILD diagnoses, potential difficulties in assessing local control outcomes due to the CT appearance of ILD exacerbations and late radiation-related lung fibrosis, as well as potentially slow accrual. We plan to open this trial at multiple international studies to accelerate the accrual process. A favourable factor for accrual is that with ASPIRE-ILD being a single-arm trial in this population of complex patients who likely have few other treatment options, enrollment in a clinical trial may become a preferred choice for clinicians.

In conclusion, patients with ES-NSCLC and co-existing ILD present a challenging therapeutic scenario due to limited available treatment options and the potential for severe treatment-related toxicity. It is our hope that the results of ASPIRE-ILD will provide clarity on the effectiveness and safety of SABR in this population of patients.

## Supplementary information

**Additional file 1.**  Summary of SABR-related mortality and ILD-specific toxicity. Reproduced from Chen et al [10].

**Additional file 2.** Consent Form for the ASPIRE-ILD Study.

**Additional file 3.** ASPIRE-MRI Sub-study (London Site Only).

**Additional file 4.** Consent Form for the ASPIRE-MRI Sub-study (London Site Only).

**Additional file 5.** Flowchart of Enrollment and Central Review.

**Additional file 6.** ASPIRE-ILD Trial Information.

## Data Availability

Not applicable.

## Abbreviations

2D: 2-dimensional
4D: 4-dimensional
AE: adverse event
AJCC: American Joint Committee on Cancer
BED: Biologically effective dose
CAPRI: Canadian Pulmonary Radiotherapy Investigators’ Group
CBCT: Cone beam computed tomography
CT: Computed tomography
CTCAE: Common Terminology Criteria for Adverse Events
CTD: Connective tissue disease
D2cm: Maximum dose at 2 cm from planning target volume as a percentage of prescription dose
DICOM: Digital Imaging and Communications in Medicine
DLCO: Diffusing capacity of the lungs for carbon monoxide
DSMC: Data safety monitoring committee
DVH: Dose-volume histogram
ECOG: Eastern Cooperative Oncology Group
EQ-5D-5 L: EuroQol 5-Dimensional 5-Level
ES-NSCLC: Early-stage non-small cell lung cancer
FACT-L: Functional Assessment of Cancer Therapy - Lung
FDG: Fluorodeoxyglucose
FEV_1_: Forced expiratory volume in 1 s
FVC: Forced vital capacity
GI: Gastrointestinal
GTV: Gross tumour volume
HRCT: High-resolution computed tomography
IAEA: International Atomic Energy Agency
ICH GCP: International Council for Harmonisation Guidelines for Good Clinical Practice
ICMJE: International Committee of Medical Journal Editors
IGTV: Internal gross tumour volume
ILD: Interstitial lung disease
ILD-GAP: Interstitial Lung Disease – Gender, Age, Physiology
IPF: Idiopathic pulmonary fibrosis
IRB: Institutional Review Board
ITV: Internal target volume
MIP: Maximal intensity projection
MRI: Magnetic resonance imaging
NSCLC: Non-small cell lung cancer
OAR: Organ at risk
OS: Overall survival
PA: Pulmonary artery
PET: Positron emission tomography
PTV: Planning target volume
PV: Pulmonary vein
QA: Quality assurance
QIPCM: Quantitative Imaging for Personalized Cancer Medicine
R100: Ratio of 100% prescription isodose volume to planning target volume
R50: Ratio of 50% prescription isodose volume to planning target volume
REB: Research Ethics Board
ROI: Region of interest
RTOG: Radiation Therapy Oncology Group
SABR: Stereotactic ablative radiotherapy
SAE: Severe adverse event
V20: Volume of lung receiving at least 20 Gy
