# Supplementary material for: Assessment of precision irradiation in early non-small cell lung cancer and interstitial lung disease (ASPIRE-ILD): study protocol for a phase II trial
Source: BMC Cancer. 2019 Dec 11;19:1206. doi: 10.1186/s12885-019-6392-8 (PMC6905060; doi:10.1186/s12885-019-6392-8)
Supplement: Supplementary file 1 — Additional file 1. Summary of SABR-related mortality and ILD-specific toxicity. Reproduced from Chen et al [10]. [file 12885_2019_6392_MOESM1_ESM.docx]

Appendix 1. Summary of SABR-related mortality and ILD-specific toxicity. Reproduced from Chen *et al.* (22)

|  | **N** | **Stage** | **Mortality Rate** | **Toxicity Rate** | **Study Design** | **ILD Diagnosis** | **BED_10_ (Gy_10_)** | **BED_3_ (Gy_3_)** |
| --- | --- | --- | --- | --- | --- | --- | --- | --- |
| Hara 2015 | 6 | I-II | 0.167 | 0.167 | Retrospective chart review | Asbestosis with subclinical IP, Unspecified IP | 72-132 | 147-300 |
| Yoshitake 2015 | 18 | I | 0.167 | 0.389 | Retrospective chart review | Interstitial changes | 106^c^ | 240^c^ |
| Jung 2015 | 1 | I | 0 | 0 | Retrospective chart review | ILD | 106-180 | 240-460 |
| Ueki 2015 | 20 | I | 0 | 0.1 | Retrospective chart review | UIP, non-UIP ILD | 105-134^c^ | 210-317^c^ |
| Shintani 2014 | 1 | I | 0 | 1 | Retrospective chart review | IP | 96-106 | 180-240 |
| Bahig 2014 | 5 | I-IIA | 0.6 | 0.6 | Retrospective chart review | IPF | 72-180 | 147-460 |
| Thibault 2014 | 1 | I | 1 | 1 | Mixed prospective database/ retrospective chart review | IPF | 106^c^ | 240^c^ |
| Aibe 2014 | 30 | I-II | 0.067 | 0.067 | Retrospective chart review | IP shadow | 100^c^ | 217^c^ |
| Yamaguchi 2013 | 16 | I^b^ | 0.062 | 0.188 | Retrospective chart review | ILD | 60-120 | 130-277 |
| Takeda 2012 | 5 | I-II | 0.2 |  | Retrospective chart review | IP | 72-100 | 147-217 |
| Yamashita 2010 | 13 | I-II | 0.538 | 0.538 | Retrospective chart review | IP shadow | 106 | 240^c^ |
| Takeda 2008 | 1 | I | 0 | 1 | Case report | Subclinical IPF | 81 | 154^c^ |
| Timmerman 2003 | 5 | I-II | 0 |  | Phase I clinical trial | Pulmonary fibrosis | 43-180 | 88-460 |

ILD: interstitial lung disease, UIP: usual interstitial pneumonia, IP: interstitial pneumonia, IPF: idiopathic pulmonary fibrosis
