# Supplementary material for: Assessment of precision irradiation in early non-small cell lung cancer and interstitial lung disease (ASPIRE-ILD): study protocol for a phase II trial
Source: BMC Cancer. 2019 Dec 11;19:1206. doi: 10.1186/s12885-019-6392-8 (PMC6905060; doi:10.1186/s12885-019-6392-8)
Supplement: Supplementary file 2 — Additional file 2. Consent Form for the ASPIRE-ILD Study. [file 12885_2019_6392_MOESM2_ESM.docx]

Appendix 2. Consent Form for the ASPIRE-ILD Study

**Stereotactic Radiation Therapy for Patients with Early Lung Cancer and Interstitial Lung Disease**

Assessment of Precision Irradiation in Early NSCLC and Interstitial Lung Disease (ASPIRE-ILD): A Phase II Trial

Study Doctor:

Sponsor: Lawson Health Research Institute, London, Canada

*If an REB approved French consent is not used at your institution remove this statement.*

Le formulaire de consentement est disponible en français sur demande.

**Emergency Contact Number** (24 hours / 7 days a week):

Non-Emergency contact numbers are at the end of this document under Contacts.

**Introduction**

You are being invited to participate in a clinical trial (a type of study that involves research). Clinical trials only include participants who choose to take part. You are being invited to participate in this study because you have an early lung cancer (called a “non-small cell lung cancer” that is less than 5 cm in size and has not spread) that cannot be surgically removed, and also because you have a chronic lung problem called interstitial lung disease. This consent form provides you with information to help you make an informed choice. Please read this document carefully and take your time in making your decision. You may find it helpful to discuss it with your friends and family.

Taking part in this study is voluntary. You may choose not to take part or if you choose to participate may leave the study at any time without giving a reason. Deciding not to take part or deciding to leave the study later will not result in any penalty or any loss of benefits to which you are entitled.

**Background**

The standard or usual treatment for your early lung cancer that cannot be surgically removed is a type of radiation treatment called “stereotactic ablative radiotherapy”, or SABR for short. SABR delivers high-dose, precise radiation in 1-3 weeks of treatments. SABR usually has mild side effects and is successful in controlling the cancer in about 8 out of 10 patients.

You have a type of lung problem called “interstitial lung disease”, or ILD for short, where the tissues of your lung show signs of inflammation and/or scarring. Previous studies showed that for participants like you, the chance that you may get a side effect where your breathing becomes worse in the months after your radiation treatment can be up to 1 in 4, which is much higher than participants without ILD. In high-risk participants, the worsening in breathing can result in death with a chance of up to 1 in 7. However, some studies have shown this risk to be much lower.

Without treatment, lung cancer can also become rapidly fatal, usually within less than a year.

The decision whether or not to have SABR rests with you and your doctor. For participants who choose to have SABR, we would like to find out long they live after treatment, and if there is a dose of SABR that can work to both cure/control your cancer while minimizing your chance of having a bad side effect on your breathing.

For participants who do not have SABR, we would like to find out how they fare without treatment.

The research ethics board, which oversees the ethical conduct of research involving humans, has reviewed and accepted this study.

**Purpose**

The purpose of this study is to:

1. Carefully determine the effects of SABR in participants with interstitial lung disease,
2. For participants who choose not to have SABR, to observe them with regular phone calls to determine how they fare.

**Alternative Treatments**

You do not have to take part in this study. Other options (in addition to the standard or usual treatment described above) may include, but are not limited to:

- Palliative care or Best Supportive Care (BSC). This type of care helps reduce shortness of breath, tiredness, appetite problems and other problems caused by the cancer. It does not treat the cancer directly, but instead tries to improve how you feel. Best Supportive Care tries to keep you as active and comfortable as possible.
- Chemotherapy, immune therapy or pill therapy. Whether these options are right for you depends on your current health and whether your cancer has special mutations that respond to pill therapy. You need to speak to a medical oncologist about these options.

Please talk to your study doctor or usual cancer doctor about the known benefits and risks of these other options before you decide to take part in this study. Your usual cancer doctor can also discuss with you what will happen if you decide not to undertake any treatment at this time.

**Expected Number of Participants**

Approximately 39 people will take part in this study and receive SABR. There is no limit on the number of participants on this study who choose not to receive SABR.

This study should take 2-3 years to complete and the results should be known in about 5 years.

Your study doctor will be informed of the results of this study once they are known.

**Assignment to a Group**

You can choose whether to have SABR or whether you would prefer not to have SABR.

**SABR Treatment**

If you agree to take part in this study and receive SABR, your treatment will take place over 2-4 weeks, with radiation treatments given every day or every other day depending on the group. There are no treatments on weekends or holidays. Each day the radiation treatment takes about 20 minutes, or sometimes longer if the study staff has to track your breathing during treatment. You will need to have a specialized computerized tomography (CT) scan to plan the radiation treatment and track your breathing movement before starting radiation treatment.

|  | **Overall dose (Gray)*** | **Number of treatments** | **Treatment frequency**** | **Total length of treatment** |
| --- | --- | --- | --- | --- |
| **Standard Dose** | **50** | **5** | **Every other day** | **2 weeks** |

*Gray/Gy is a unit of radiation. Even though the overall dose is the same, decreasing the number of treatments makes the effective dose to your cancer, and also your lung, higher.

**except weekends and holidays

If there are unacceptable side effects using the standard dose of 50 Gy in 5 treatments for some participants in the study, the treatment will be made more gentle for future participants. This is done by giving the radiation in smaller amounts each day, spreading it over 10 days or 15 days, not including weekends. This gentler approach might reduce the side effects but might also reduce the chances of curing the cancer. You will be told if you are receiving a dose that is different than the standard dose.

**Observation**

If you choose not to have SABR, you and your doctors will decide on which medical options are best for you. You will not need any more visits as part of this study. Study staff will call you every 3 months for two years to see if you are having breathing problems. This call will take about 20 minutes. Study staff will also use the information in your medical records to learn how you are doing.

**Study Procedures**

**Non-Experimental Procedures**

The following tests and procedures will be done as part of this study. Some of these tests and procedures may be done as part of your standard care, in which case the results may be used. Some of these tests may be done more frequently than if you were not taking part in this study and some may be done solely for the purpose of the study. If the results show that you are not able to continue participating, your study doctor will let you know:

- Physical examination
- High-resolution computerized tomography (CT) scan of your chest. A CT scan is a series of x-rays of the body from many angles that are turned into 3-dimensional pictures on a screen. CT scans often involve injecting a dye into your vein.
- Positron emission tomography (PET) scan of your whole body. A PET scan is a scan to help show how organs and tissues are working by tracing where a small amount of glucose (a sugar) that includes a tiny, harmless amount of radioactivity, goes in your body after it has been injected into one of your veins.
- Spirometry/pulmonary function test. You will be asked to breath into a machine in order to measure how well your lungs work. Some blood may also be taken to adjust the values for your blood counts.

The follow tests may be required if your cancer doctors think that they are necessary, if they have not already been done:

- Sampling of the lymph nodes in your chest. This may be done as a short surgery or with an endoscope (a camera and collection of tools in a thin tube that goes into your lung through your mouth or nose).
- Biopsy of your lung cancer. This may be done with a needle through your chest guided by CT or with an endoscope.
- Magnetic resonance imaging (MRI) scan of your brain. An MRI scan is a scan that uses a strong magnet to produce pictures of areas inside the body*.* The scan will involve injecting a dye into your vein.

If the results of the test(s) show that you are not able to continue participating, your doctor will let you know.

**Questionnaires**

If you receive SABR, you will be provided with a questionnaire before starting this study, and after your treatment finishes you will be asked to fill out the same questionnaire every 3 months for one year, then every 6 months for one year, then every year for three years. The purpose of the questionnaire is to understand how your treatment and illness affects your quality of life. Each questionnaire will take about 15 minutes to complete. It is an important part of the study to complete the questionnaires regularly.

The information you provide is for research purposes only and will remain strictly confidential.

Some of the questions are personal; you may choose not to answer these if you wish.

Even though you may have provided information on a questionnaire, these responses will not be reviewed by your health care team or study team. If you wish them to know this information please bring it to their attention.

**Central Clinical, Radiological and Pathological Review**

Copies of your treatment plan and imaging scans will be collected as part of this study. This is required for quality assurance and data management for research. The copies will be sent to Quantitative Imaging for Personalized Cancer Medicine (QIPCM), based in Toronto Ontario, stored in a secure file, and kept until the end of the study monitoring period*,* when then they will be destroyed. QIPCM provides centralized storage and data analysis tools for medical imaging, and is compliant with privacy regulations.

Copies of your high-resolution CT scan images and reports, lung pathology slides and reports (if available), and clinical information (via a questionnaire your doctor fills out) will be collected as part of this study. This is required to decide whether this study is right for you and for quality assurance. Copies of images, reports and questionnaires will be double-checked by a group of experts outside London, Ontario. Pathology slides will be returned to the hospital where they came from.

To protect your identity, the information that will be on your reports and questionnaires will be limited to your study identification number and partial initials.

*The following paragraph is for London participants only. All other Centres are to delete the following paragraph.*

**Optional Magnetic Resonance Imaging (MRI) Scans**

The researchers doing this study are interested in doing additional research to see how focused radiation therapy (stereotactic ablative radiotherapy, or SABR) affects your lungs’ ability to breathe and process oxygen by doing special magnetic resonance imaging (MRI) scans. You will be given an additional optional study consent form to read and sign if you wish to give permission to have these additional scans. You may decide not to participate in the "optional" study and still participate in this main study.

**Responsibilities**

If you choose to participate in this study, you will be expected to:

- Tell your study doctor about your current medical conditions;
- Tell your study doctor about all prescription and non-prescription medications and supplements, including vitamins and herbals;
- Tell your study doctor if you are thinking about participating in another research study;
- Complete the prescribed radiation treatment;
- Complete all recommended tests;
- Attend all scheduled follow-up appointments;
- Complete all questionnaires when returning for follow-up appointments;
- Tell your study doctor if you become pregnant

**Length of Participation**

**If you choose SABR**, your radiation treatment will last for about 2 weeks.

You will be asked to come back to the clinic 3 months after finishing your radiation treatment, then every 3 months until 1 year after finishing your treatment. You will then be asked to come back to the clinic every 6 months until 2 years after finishing your treatment, and then yearly until 5 years after finishing your treatment. This is similar to the usual recommended frequency of follow-up appointments after SABR. At each clinic visit, study staff will ask you some questions to see if you have any side effects from the radiation treatment. Study staff will also ask you to fill out questionnaires at each clinic visit to see how the illness and treatment has affected your quality of life.

You will have repeat high-resolution CTs of your chest to see how the cancer is responding to the radiation treatment, and to see whether the lungs show any signs of radiation damage. CTs will be scheduled at 3, 6, 12, 18, and 24 months after your treatment, and then yearly until 5 years after finishing your treatment. This is similar to the usual recommended frequency of CTs following SABR.

In addition to seeing your cancer doctor, you will see a doctor that specializes in ILD (respirologist or pulmonologist) every 6 months for 1 year after finishing your treatment, and then yearly until 5 years after finishing your treatment. You will also have repeat pulmonary function tests to see how well your lungs function every 6 months for 2 years after finishing your treatment, and then yearly until 5 years after finishing your treatment.

You may be seen more often if your study doctor determines that this is necessary, or if your cancer shows signs of coming back.

**If you choose not to have SABR,** you will not need any more visits as part of this study. Study staff will call you every 3 months for two years to see if you are having breathing problems. This call will take about 20 minutes.

**Early End to Participation**

Your participation in the trial may be stopped early, for reasons such as:

- You are unable to tolerate the study treatment.
- You are unable to complete all required study procedures.
- Your study doctor no longer feels this is the best treatment for you.
- A Regulatory authority such as the Research Ethics Board withdraws permission for the study to continue.

If your participation in the study is stopped your study doctor will provide information about how to stop safely.

**Risks of SABR**

Participating in this study is not expected to put you at higher risk than the standard of care SABR treatment. If you choose to take part in this study, there is a risk that:

- You may lose time at work or home and spend more time in the cancer centre than usual
- You may be asked sensitive or private questions which you normally do not discuss
- You may receive a lower dose of radiation than you otherwise would have received with standard of care treatment. It is possible that this can reduce the chance that your cancer is cured or controlled.

The radiation treatment used in this study may affect how different parts of your body work such as your lungs, heart and esophagus (swallowing tube).

Here are important points about side effects:

- The study doctors do not know who will or will not have side effects.
- Some side effects may go away soon, some may last a long time, or some may never go away.
- Some side effects may be serious and may even result in death.

Here are important points about how you and the study doctor can make side effects less of a problem:

- Tell the study doctor if you notice or feel anything different so they can see if you are having a side effect.
- The study doctor may be able to treat some side effects.

The tables below show the most common and the most serious side effects that researchers know about. There might be other side effects that researchers do not yet know about. If important new side effects are found, the study doctor will discuss these with you.

| COMMON, SOME MAY BE SERIOUS  In 100 people receiving radiation therapy, more than 20 and up to 100 may have: |
| --- |
| - Reddening or tanning of the skin - Tiredness - Dry cough |

| OCCASIONAL, SOME MAY BE SERIOUS  In 100 people receiving radiation therapy, from 4 to 20 may have: |
| --- |
| - Sore throat or difficulty swallowing - A mild worsening of shortness of breath - A fast, severe worsening of shortness of breath caused by the radiation treatment, which can lead to death |

| RARE, AND SERIOUS  In 100 people receiving radiation therapy, 3 or fewer may have: |
| --- |
| - Damage to internal organs, such as the spinal cord, esophagus (swallowing tube), lungs, heart, bones and nerves. The damage may be permanent |

Let your study doctor know of any questions you have about possible side effects. You can ask the study doctor questions about side effects at any time.

**Reproductive Risks**

The effects that the radiation may have on an unborn baby (fetus) are unknown. You must not become pregnant or father a baby while taking part in this study.

You must not become pregnant or father a baby while receiving radiation treatment and for 1 year after the last treatment. If you become pregnant or father a child during this study or for 1 year after the last radiation treatment, then you should immediately notify your study doctor.

Your study doctor will discuss methods with you to ensure that you do not become pregnant or father a baby during the study.

# **Data Safety Monitoring Board/Committee**

A Data Safety Monitoring Board/Committee, an independent group of experts, will be reviewing the data from this research throughout the study.

**Benefits**

If you agree to receive SABR, it will likely still be an effective treatment to cure or control your cancer. The chances that your cancer will not come back is higher with the study treatment than receiving no treatment, best supportive care or chemotherapy/pill therapy. You will also be monitored closely during and after your treatment to check on both your cancer and how well you breathe.

The researchers hope the information learned from this study will help other patients in the future.

**Confidentiality**

Records identifying you at this centre will be kept confidential and, to the extent permitted by the applicable laws, will not be disclosed or made publicly available, except as described in this consent document.

Authorized representatives of the following organizations may look at your original (identifiable) medical/clinical study records at the site where these records are held, for quality assurance (to check that the information collected for the study is correct and follows proper laws and guidelines):

- The Ontario Cancer Research Ethics Board, which oversees the ethical conduct of this study in your clinic/hospital;
- The Canadian Pulmonary Radiotherapy Investigators Group
- Governmental agencies that supervise clinical research, such as Health Canada

Authorized representatives of the following organizations may **receive** information related to the study from your medical/clinical study records for quality assurance and data analysis. Your name or other information that may identify you will not be used. The records received by these organizations may contain your study identification code and initials:

- Central review centre in London, Canada

All of the organizations listed in the above confidentiality sections are required to have strict policies and procedures to keep the information they see or receive about you confidential, except where disclosure may be required by law. The study doctor will ensure that any personal health information collected for this study is kept in a secure and confidential location as required by law. There are federal and provincial laws that these organizations must comply with to protect your privacy.

If the results of this study are published, your identity will remain confidential. It is expected that the information collected during this study will be used in analyses and will be published/ presented to the scientific community at meetings and in journals.

Even though the likelihood that someone may identify you from the study data is very small, it can never be completely eliminated.

A copy of this signed and dated consent form may be included in your health record/hospital chart.

Your family doctor/health care provider will be informed that you are taking part in a study so that you can be provided with appropriate medical care. If you do not want you family doctor/health care provider to be informed, please discuss with your study doctor.

A wallet card will be provided to you with information about how to contact the study staff when required

Your de-identified data from this study may be used for other research studies. If your study data is shared with other researchers, information that links your study data directly to you will not be shared.

**Registration of Clinical Trials**

A description of this clinical trial will be available on <http://www.clinicaltrials.gov>, as required by U.S. Law. This Web site will not include information that can identify you. At most, the Web site will include a summary of the results. You can search this Web site at any time.

**Costs**

The costs of your radiation treatment will be paid for by your provincial medical plan to the extent that such coverage is available.

Taking part in this study may result in added costs to you (i.e. transportation, parking, meals, or unpaid leave from work). You may have to pay for medication prescribed to treat or prevent side effects, and you may have to visit the hospital more often than if you were not participating in this study.

**Compensation**

You will not be paid for taking part in this study.

You will be reimbursed for study-related expenses such as specify, e.g., parking, etc.

Note: this statement may be removed/revised as per centre requirements.

In the case of research-related side effects or injury, medical care will be provided by your doctor or you will be referred for appropriate medical care.

**Rights**

You will be told, in a timely manner, about new information that may be relevant to your willingness to stay in this study.

If you decide to stop participating in the study or if your participation has been stopped, your doctor will discuss other options with you and continue to treat you with the best means available.

You may withdraw your permission to use your personal health information for this study at any time by letting the study doctor know. However, this would also mean that you withdraw from the study. Your study data that was recorded before you withdrew will be used but no information will be collected or sent to the sponsor after you withdraw your permission.

Your rights to privacy are legally protected by federal and provincial laws that require safeguards to ensure that your privacy is respected.

By signing this form you do not give up any of your legal rights against the investigators, sponsor or involved institutions for compensation, nor does this form relieve the investigators, sponsor or involved institutions of their legal and professional responsibilities.

You will be given a copy of this signed and dated consent form prior to participating in this study.

**Conflict of Interest**

This study is funded partially by a philanthropic donation and also supported by a grant from the Ontario Institute for Cancer Research through funding provided by the Government of Ontario. The researchers at this centre will not receive any direct benefit for conducting this study.

The doctor treating you also may be the doctor in charge of the study.

If you would like additional information about the funding for this study, or about the role of the doctor in charge of this study, please speak to the study staff or to the Office of the Chair of the Ontario Cancer Research Ethics Board. (contact information below)

**Contacts**

If you have questions about taking part in this study, or if you suffer a research-related injury, you should talk to your study doctor. Or, you can meet with the doctor who is in charge of the study at this institution. That person is:

| Name: |  | Telephone: |
| --- | --- | --- |

If you have questions about your rights as a participant or about ethical issues related to this study, you can talk to someone who is not involved in the study at all. Please contact the Office of the Chair of the Ontario Cancer Research Ethics Board at:

| Telephone: 416-673-6648 |  | Toll Free: 1-866-678-6427 ext. 6648 |
| --- | --- | --- |

**Signatures**

- All of my questions have been answered,
- I understand the information within this informed consent form,
- I allow access to my medical records as explained in this consent form,
- I am aware of the risks to me of participating in the study and the risks to the fetus if I become pregnant or father a child during this study,
- I do not give up any of my legal rights by signing this consent form,
- I agree to take part in this study

**Choose one option:**

1. **I choose to have SABR** ____________

(initial)

1. **I choose to be observed and not have SABR** ____________

(initial)

| Signature of Participant |  | Printed Name |  | Date |
| --- | --- | --- | --- | --- |

| Signature of Person Conducting the Consent Discussion |  | Printed Name |  | Date |
| --- | --- | --- | --- | --- |

**Participant Assistance**

**Complete the following declaration only if the participant is unable to read:**

- The informed consent form was accurately explained to, and apparently understood by, the participant, and
- Informed consent was freely given by the participant

| Signature of Impartial Witness |  | Printed Name |  | Date |
| --- | --- | --- | --- | --- |

**Complete the following declaration only if the participant has limited proficiency in the language in which the consent form is written and interpretation was provided as follows:**

- The informed consent discussion was interpreted by an interpreter, and
- A sight translation of this document was provided by the interpreter as directed by the research staff conducting the consent.

**Interpreter Declaration and Signature:**

By signing the consent form I attest that I provided a faithful interpretation for any discussion that took place in my presence, and provided a sight translation of this document as directed by the research staff conducting the consent.

| Signature of Interpreter |  | Printed Name |  | Date |
| --- | --- | --- | --- | --- |
