# Supplementary material for: Assessment of precision irradiation in early non-small cell lung cancer and interstitial lung disease (ASPIRE-ILD): study protocol for a phase II trial
Source: BMC Cancer. 2019 Dec 11;19:1206. doi: 10.1186/s12885-019-6392-8 (PMC6905060; doi:10.1186/s12885-019-6392-8)
Supplement: Supplementary file 3 — Additional file 3. ASPIRE-MRI Sub-study (London Site Only). [file 12885_2019_6392_MOESM3_ESM.docx]

Appendix 3: ASPIRE-MRI Substudy (London Site Only)

Patients who consent to this substudy will undergo 129-Xe MRI at Robarts Research Institute at 2 time-points: pre-SABR, 6-months post-SABR.

**Objectives**

To assess changes in pulmonary ventilation in patients with ILD who have undergone SABR using 129-Xe MRI.

**Imaging Protocol Details:**

Xenon-129 MRI will be acquired in patients with Interstitial Lung Disease (ILD) as a part of the ASPIRE-ILD study. Four image sets will be acquired within a 30 minute imaging session at Robarts Research Institute:

- Series 1: 3 plane localizer – for patient localization
- Series 2: Bag matched ^1^H MRI – an anatomical reference for the ^129^Xe images
- Series 3: ^129^Xe MR multiple-b apparent diffusion coefficient (ADC) image – to probe lung microstructure
- Series 4: ^129^Xe MR static ventilation (SV) image – to determine the ventilation defect percent (VDP)

The image acquisition protocols are listed below.

**Series 1:** Three plane localizer

**Series 2:** ^1^H MRI bag matched 1.0L of N_2_

| **Imaging Parameters** | |
| --- | --- |
| Orientation | Coronal |
| Coil Type | Body Coil |
| PSD type | 2D Multi-slice |
| FOV (cm^2^) | 40x40 |
| Flip angle (^◦^) | 30 |
| Matrix Size | 128x80 (zero-padded to 128x128) |
| Bandwidth (kHz) | 24.4 |
| TE (ms) | 1.2 |
| TR (ms) | 4.7 |
| Slice thickness (mm) | 15 |
| Spacing | 0 |
| Number of slices | 15 |
| Acquisition time (s) | 15 |

**Series 3:** ^129^Xe MRI Multiple-b ADC using 1.0L bag of hyperpolarized ^129^Xe/^4^He (0.5L/0.5L)

| **Imaging Parameters** | |
| --- | --- |
| Orientation | Coronal |
| Coil Type | ^129^Xe Birdcage Coil |
| PSD type | 3D FGRE |
| FOV (cm^2^) | 40x30 |
| Flip angle (^◦^) | Variable |
| Matrix Size | 128x94 (zero-padded to 128x128) |
| Bandwidth | 25 |
| TE (ms) | 10 |
| TR (ms) | 11 |
| b-value (s/cm^2^) | 0, 12, 20, 30, 40 |
| Slice thickness (mm) | 30 |
| Spacing | 0 |
| Number of slices | 7 |
| Acquisition time (s) | 16 |

**Series 4:** ^129^Xe MRI SV using 1.0L bag of hyperpolarized ^129^Xe/^4^He (0.5L/0.5L)

| **Imaging Parameters** | |
| --- | --- |
| Orientation | Coronal |
| Coil Type | ^129^Xe Birdcage Coil |
| PSD type | 3D FGRE |
| FOV (cm) | 40x40 |
| Flip angle (^◦^) | Variable |
| Matrix Size | 128x80 (zero-padded to 128x128) |
| Bandwidth | 9 |
| TE (ms) | 1.8 |
| TR (ms) | 7 |
| Slice thickness (mm) | 15 |
| Spacing | 0 |
| Number of slices | 15 |
| Acquisition time (s) | 13 |

**Image Analysis:**

**1)** *Apparent Diffusion Coefficients (ADC):*

During a single breath-hold, two interleaved images are acquired – one without diffusion sensitization and the second image acquired with the presence of diffusion sensitization through the application of the additional pulsed magnetic field gradient with pulse duration and maximum value **g**. The signal intensity of the second image is derived from:

|  |  |  |
| --- | --- | --- |

where is a signal intensity of the first image, ADC is an apparent self-diffusion coefficient and b =  ( is a gyromagnetic ratio). Accordingly, the ADC value for each image pixel on pixel by pixel basis or location can be calculated from the following equation:

|  |  |  |
| --- | --- | --- |

Diffusion weighted images from each visit will be reviewed and compared with the ^1^H anatomical images. Mean, whole lung, and centre slice ADC (and standard deviation) will be calculated from the maps based on the formula above and recorded for both visits. ADC values will also be calculated for each slice and for six regions of interest (ROI) to assess regional variation in this measure. ADC gradients in the superior-inferior and anterior-posterior directions will be calculated for each subject and visit.

**2)** *Mean Linear Intercept (L_m_):*

The signal dependence related to diffusion-sensitization can be determined through the probability density function or diffusion propagator (*P*) for fluid diffusion in confined media with unknown geometry (1-3):

|  |  | (1) |
| --- | --- | --- |

where *D* is diffusivity, *S*(b) is the signal at a particular b-value and *S_0_* is the MR signal-intensity in the absence of diffusion-sensitizing gradients. The diffusion propagator can be ascertained through the inverse Laplace transform of *S*(b) (2) and to apply this, the analytical representation for *S*(b) is required. Thus, experimental *S*(b) values can be fit as demonstrated for multi-b diffusion-weighted ^3^He MRI (3-5) as follows:

|  |  | (2) |
| --- | --- | --- |

where is the apparent diffusivity and *α* is the heterogeneity index (0<α≤1.0). The diffusion propagator can be determined through substitution of Eqn. [2] into Eqn. [1] and then applying the inverse Laplace transform (2):

|  |  | | | (3) |
| --- | --- | --- | --- | --- |
| And | |  | (4) | |

where f(*D*) is the auxiliary function and parameters *B* and *C* are functions of the heterogeneity index (2). Mean *D* estimates can be determined using the probability density function distribution to calculate mean airway length maps (1) (*Lm*_D_ = $\sqrt{2\Delta D}$, where $\Delta is the diffusion time$). For multi-b diffusion-weighted ^3^He MRI, *L*_m_ is empirically observed to be proportional to *Lm*_D_ (3):

 (5)

Mean airway length depends on both Δ and diffusivity, so Eqn. [5] cannot be used for ^129^Xe MRI-based *L*_m_ estimates. In order to extend Eqn. [5] to ^129^Xe gas, the empirical relationship in Equation 6 was previously determined and proposed (6):

 (6)

where is the free diffusion coefficient of ^3^He (0.88 cm^2^/s), Δ_He_=1.46 ms (3), is the free diffusion coefficient of ^129^Xe (0.12 cm^2^/s) and Δ_Xe_ is the diffusion time.

**3)** *Ventilation Defect Percent (VDP):*

Ventilation defect volume (VDV), ventilation defect percent (VDP), ventilation volume (VV) and percent ventilated volume (PVV) will be generated for whole lung and individual lung slices using manual or semi-automated segmentation. For ^1^H thoracic cavity images, the thoracic cavity volume (TCV) will be calculated by semi-automated segmentation (7).

**Patient Compensation**

Patients will be compensated $50 CDN for each of two visits to Robarts Research Institute (one pre-treatment, and one post-treatment). This is similar to procedures from previous studies (e.g. the Functional Lung Avoidance for Individualized Radiotherapy [FLAIR] trial, NCT02002052)

**References for Appendix 2**

1. Parra-Robles J, Marshall H, Hartley RA, Brightling CE, Wild JM. Quantification of lung microstructure in asthma using a 3He fractional diffusion approach [abstract]. ISMRM 22nd Annual Meeting. 2014:3529.

2. Berberan-Santos MN, Bodunov EN, Valeur B. Mathematical functions for the analysis of luminescence decays with underlying distributions 1. Kohlrausch decay function (stretched exponential). Chem Phys. 2005;315(1-2):171-82.

3. Ouriadov A, Lessard E, Sheikh K, Parraga G, for the Canadian Respiratory Research N. Pulmonary MRI morphometry modeling of airspace enlargement in chronic obstructive pulmonary disease and alpha-1 antitrypsin deficiency. Magn Reson Med. 2017.

4. Chan HF, Stewart NJ, Parra-Robles J, Collier GJ, Wild JM. Whole lung morphometry with 3D multiple b-value hyperpolarized gas MRI and compressed sensing. Magn Reson Med. 2016.

5. Abascal JFPJ, Desco M, Parra-Robles J. Incorporation of prior knowledge of the signal behavior into the reconstruction to accelerate the acquisition of MR diffusion data. ArXiv e-prints [serial online]. 2017; vol 1702. Available at: http://adsabs.harvard.edu/abs/2017arXiv170202743A.

6. Ouriadov A, Lessard E, Guo F, et al. Accelerated Diffusion-weighted 129Xe MRI Morphometry of Emphysema in COPD and Alpha-1 Antitrypsin Deficiency Patients [abstract]. ISMRM 25th Annual Meeting, Honolulu, Hawaii, USA. 2017([abstract]):1763.

7. Kirby M, Heydarian M, Svenningsen S, et al. Hyperpolarized 3He magnetic resonance functional imaging semiautomated segmentation. Academic radiology. 2012;19(2):141-52.
