# Supplementary material for: Assessment of precision irradiation in early non-small cell lung cancer and interstitial lung disease (ASPIRE-ILD): study protocol for a phase II trial
Source: BMC Cancer. 2019 Dec 11;19:1206. doi: 10.1186/s12885-019-6392-8 (PMC6905060; doi:10.1186/s12885-019-6392-8)
Supplement: Supplementary file 4 — Additional file 4. Consent Form for the ASPIRE-MRI Sub-study (London Site Only). [file 12885_2019_6392_MOESM4_ESM.docx]

Appendix 4. Consent Form for the ASPIRE-MRI Substudy (London Site Only)

**Informed Consent Form for Taking Part in Optional Research**

**Focused Radiation Therapy for Patients with Early Lung Cancer and Interstitial Lung Disease – Magnetic Resonance Imaging (ASPIRE-MRI)**

Study Doctor:

Sponsor: Lawson Health Research Institute, London Ontario

*If an REB approved French consent is not used at your institution you should remove the above statement.*

Le formulaire de consentement est disponible en français sur demande.

**INTRODUCTION**

In addition to the main study, you also are being invited to take part in optional research. Although it is optional, the study of exactly how radiation therapy affects the lungs of patients with interstitial lung disease is very important. Taking part in this optional research is voluntary. You still can take part in the main study, and will continue to receive treatment and care, even if you say “no” to this optional research now or later. This form and your discussion with the researcher/research staff will give you the information you need to make your decision.

**PURPOSE**

The researchers doing this study are interested in seeing how focused radiation therapy (stereotactic ablative radiotherapy, or SABR) affects your lungs’ ability to breathe and process oxygen by doing special magnetic resonance imaging (MRI) scans. An MRI scan is a scan that uses a strong magnet to produce pictures of areas inside the body. A special gas (Xenon-129) is used to make your lungs better seen on MRI scans.

**STUDY PROCEDURES**

We will ask that you receive two MRI scans as part of this optional study. The first scan will be before the start of your radiation treatment, the second will be 6 months after the end of your radiation treatment,. Each scan is expected to take about 30 minutes. You will not need an injection of dye for these MRI scans. You will be asked to breathe a special gas, called Xenon-129, as part of these MRI scans. Xenon-129 is a naturally-occuring element and it is not radioactive. Xenon-129 is important in making your lungs better seen with MRI scans.

**RISKS OF PARTICIPATION**

We will ask you questions to see if you can have the MRI scan. If you are anxious in small, tight places, having an MRI can make you feel anxious. The Xenon-129 gas does not remain in the body. However, some patients may have feelings of temporary dizziness and sensation changes (including euphoria – a strong feeling of happiness) after breathing Xenon-129, and these feelings usually last for a few minutes. In rare situations, the MRI scan can pick up an unexpected abnormality in the body that may not have been picked up otherwise, leading to possible anxiety while the abnormality is investigated. The additional time required to participate may be burdensome to some patients.

**BENEFITS**

You will not benefit directly from taking part in this optional research study. Researchers might make discoveries that could benefit people in the future.

**CONFIDENTIALITY**

Your privacy is very important to the researchers and they will make every effort to protect it.

Here are just a few of the steps they will take:

- When your MRI scans are sent to the Robarts Research Institute, no information identifying you (such as your name) will be sent. The scans may be identified by your study code, and the date the scan was performed.
- The list that links the scans to your name will be kept separate from your scan and health information in a secure and confidential location at the study site. If you change your mind about participating in this research, this list will be used to locate your samples.
- A record of your participation in this optional study will be kept with your main study records and may be monitored for quality assurance.

Information that identifies you will be kept confidential and, to the extent permitted by the applicable laws, will not be disclosed or made publicly available. If research results are published, your name and other personal information will not be used.

**WHAT IF RESEARCHERS DISCOVER SOMETHING ABOUT YOU?**

During the study, the researchers may learn something about you that they didn’t expect. For example, the researchers may see a new abnormal enlargement of your main blood vessel, the aorta, on an MRI scan that otherwise may not have been seen.

If any new clinically important information about your health is obtained as a result of your participation in this optional research, your study doctor will discuss with you the meaning of the results and if any further tests or procedures will have to be done.

**COSTS AND COMPENSATION**

You will be compensated $50 Canadian for each of the two visits to the Robarts Research Institute in London, Canada to get the MRI scans. This is to help with the costs of travel and parking.

It is possible that the research conducted using your study data may eventually lead to the development of new diagnostic tests, new drugs or other commercial products. There are no plans to provide payment to you if this happens.

**RIGHTS**

You will be told, in a timely manner, about new information that may be relevant to your willingness to stay in this study.

If you decide that you no longer want to participate, you should tell your study doctor. You will not be asked to have any further MRI scans. However, existing scans collected before you withdrew will be analyzed and the results will be reported without any identifying information from you.

You will be given a copy of this signed and dated consent form prior to participating in this study.

**CONFLICT OF INTEREST**

This study is funded partially by a philanthropic donation and also supported by a grant from the Ontario Institute for Cancer Research through funding provided by the Government of Ontario. The researchers at this centre will not receive any direct benefit for conducting this study.

The doctor treating you also may be the doctor in charge of the study.

**CONTACTS**

If you have questions about the use of your samples for research, or if you suffer a research-related injury, contact the study doctor:

_____________________________________ _________________________

Name Telephone

If you have questions about your rights as a participant or about ethical issues related to this study, you can talk to someone who is not involved in the study at all. Please contact the Office of the Chair of the Ontario Cancer Research Ethics Board at: 416-673-6648 OR,

Toll free: 1-866-678-6427 ext. 6648.

**Consent to take part in this optional research**

Please circle your answer to show whether or not you would like to take part in each option:

**For the ASPIRE-MRI study**

I agree to participate in the optional research study using Xenon-129 MRI scans as decribed above.

YES NO

**Future Contact**

I agree that my study doctor, or their representative, may contact me or my physician to see if I wish to learn about results from this research.

YES NO

**SIGNATURES**

- All of my questions have been answered,
- I understand the information within this optional consent form,
- I allow access to my medical records as explained in this consent form,
- I do not give up any of my legal rights by signing this consent form,
- I agree to take part in this study where I circled “YES”.

____________________________ ________________________ _________________

Signature of participant Printed name Date

____________________________ ________________________ _________________

Signature of person conducting Printed name Date

the consent discussion

Participant Assistance

**Complete the following declaration only if the participant is unable to read:**

- The informed consent form was accurately explained to, and apparently understood by, the participant, and
- Informed consent was freely given by the participant

____________________________ ________________________ _________________

Signature of impartial witness Printed name Date

**Complete the following declaration only if the participant has limited proficiency in the language in which the consent form is written and interpretation was provided as follows:**

- The informed consent discussion was interpreted by an interpreter, and
- A sight translation of this document was provided by the interpreter as directed by the research staff conducting the consent.

INTERPRETER DECLARATION AND SIGNATURE:

By signing the consent form I attest that I provided a faithful interpretation for any discussion that took place in my presence, and provided a sight translation of this document as directed by the research staff conducting the consent.

____________________________ ________________________ _________________

Signature of interpreter Interpreter printed name Date
