# Supplementary figures and images for: Assessment of precision irradiation in early non-small cell lung cancer and interstitial lung disease (ASPIRE-ILD): study protocol for a phase II trial
Source: BMC Cancer. 2019 Dec 11;19:1206. doi: 10.1186/s12885-019-6392-8 (PMC6905060; doi:10.1186/s12885-019-6392-8)

Appendix 5. Flowchart of Enrollment and Central Review


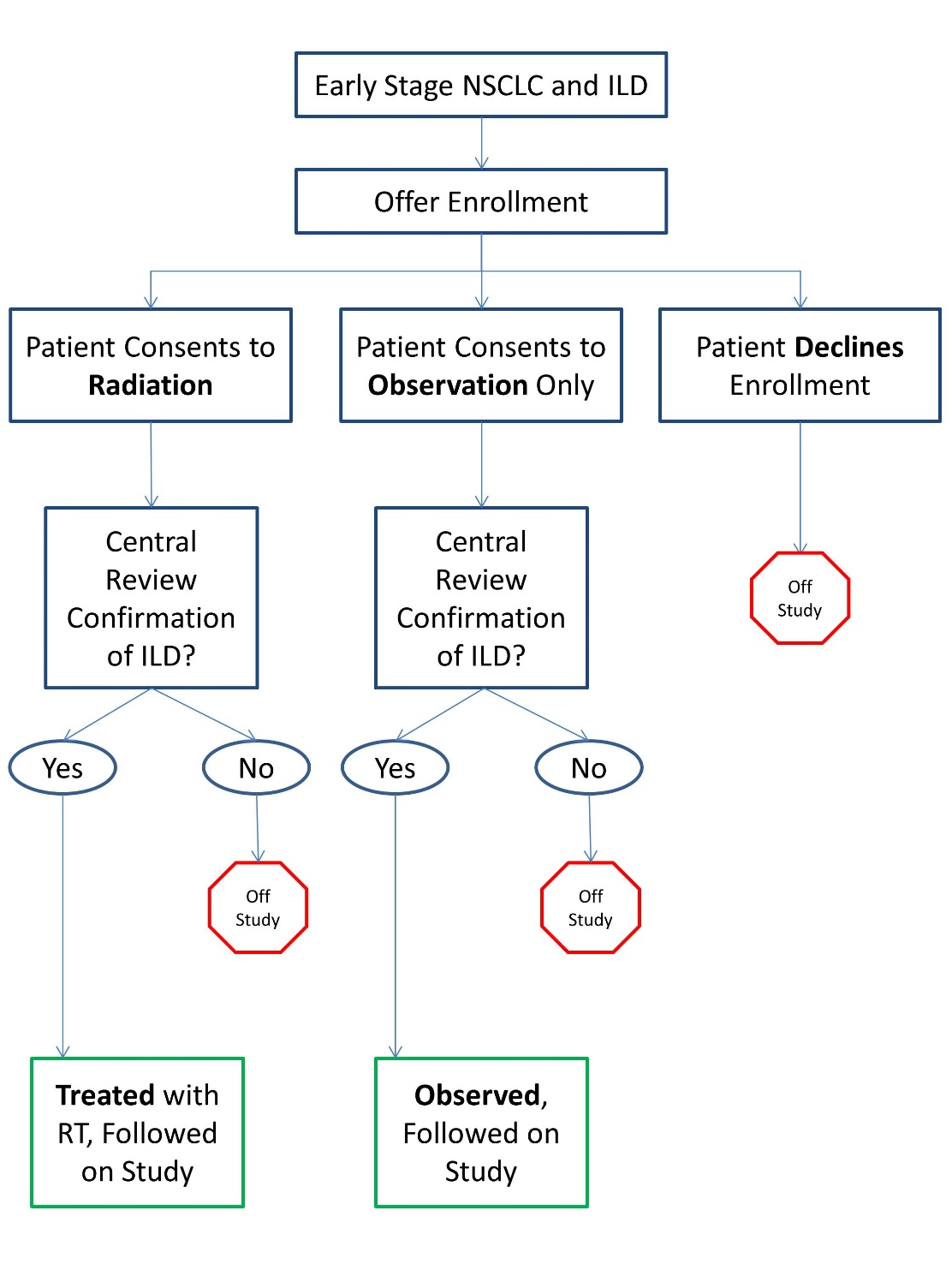

Supplement: Supplementary file 5 — Additional file 5. Flowchart of Enrollment and Central Review. [file 12885_2019_6392_MOESM5_ESM.docx]
