# Supplementary material for: Assessment of precision irradiation in early non-small cell lung cancer and interstitial lung disease (ASPIRE-ILD): study protocol for a phase II trial
Source: BMC Cancer. 2019 Dec 11;19:1206. doi: 10.1186/s12885-019-6392-8 (PMC6905060; doi:10.1186/s12885-019-6392-8)
Supplement: Supplementary file 6 — Additional file 6. ASPIRE-ILD Trial Information. [file 12885_2019_6392_MOESM6_ESM.docx]

**Appendix 6. ASPIRE-ILD Trial Information**

| Primary registry and trial identifying number | ClinicalTrials.gov NCT03485378 |
| --- | --- |
| Date of registration in primary registry | April 2, 2018 |
| Secondary identifying numbers | N/A |
| Source(s) of monetary or material support | Lawson Health Research Institute |
| Primary sponsor | Lawson Health Research Institute  750 Base Line Road East, Suite 300 London, Ontario, Canada N6C 2R5  **Phone**: 519-646-6005 **Facsimile**: 519-432-7367 **Email**: info@lawsonresearch.com |
| Secondary sponsor(s) | N/A |
| Contact for public queries | David Palma, MD, MSc, PhD  +1 519 685-8500  david.palma@lhsc.on.ca |
| Contact for scientific queries | David Palma, MD, MSc, PhD  +1 519 685-8500  david.palma@lhsc.on.ca |
| Public title | Assessment of Precision Irradiation in Early Non-Small Cell Lung Cancer and Interstitial Lung Disease (ASPIRE-ILD): Study Protocol for a Phase II Trial |
| Scientific title | Assessment of Precision Irradiation in Early Non-Small Cell Lung Cancer and Interstitial Lung Disease (ASPIRE-ILD): Study Protocol for a Phase II Trial |
| Countries of recruitment | Academic centres in Canada (current) and Europe (future) |
| Recruiting centres | - London Health Sciences Centre   - 790 Commissioners Road East, London, Ontario, Canada N6A 4L6   - Ethics approval obtained   - Study open - Princess Margaret Cancer Centre   - 610 University Ave, Toronto, Ontario, Canada M5G 2C1   - Ethics approval obtained   - Study open - Odette Cancer Centre   - 2075 Bayview Avenue T-wing, Toronto, Ontario, Canada M4N 3M5   - Ethics approval obtained   - Study open |
| Health condition(s) or problem(s) studied | Lung cancer, Interstitial lung disease |
| Intervention(s) | Stereotactic ablative radiotherapy |
| Key inclusion and exclusion criteria | - Inclusion: - Stage T1-2, N0, M0 (AJCC Staging, 8th Edition - i.e. tumor size ≤ 5 cm) prior to registration. - Not a candidate for surgical resection, determined by any of the following: - Consultation with a thoracic surgeon - Discussion at Multidisciplinary Team (MDT) rounds with a surgeon present - Patient refusal of surgery - Pathologically (histologically or cytologically) proven diagnosis of non-small cell lung cancer (NSCLC) is not required, but strongly recommended. - If the risk of biopsy is unacceptable, pathologic confirmation is not required providing there is growth over time on Computed Tomography (CT) imaging and/or Fluorodeoxyglucose (FDG) avidity that is strongly suggestive of a primary NSCLC. - Eastern Cooperative Oncology Group (ECOG) performance status 0-3; - Age ≥ 18; - Life expectancy > 6 months - Fibrotic interstitial lung disease of any subtype, as diagnosed by a respirologist/pulmonologist. - Exclusion: - Prior invasive malignancy (except non-melanomatous skin cancer) unless disease free for a minimum of 2 years (e.g., carcinomas in situ of the breast, oral cavity, or cervix are permissible); previous lung cancer, if the patient is disease-free for a minimum of 2 years is permitted. - Prior thoracic radiotherapy - Plans for the patient to receive other local therapy while on this study, except at disease progression; - Plans for the patient to receive systemic therapy (including standard chemotherapy or biologic targeted agents), while on this study, except at disease progression. Patients are allowed to receive anti-fibrotic agents used in the treatment of IPF or non-IPF fibrotic ILD (e.g. nintedanib, pirfenidone), or steroids, if those are part of their current ILD treatment regimen. Other immunosuppressive drugs such as mycophenolate, azathioprine, cyclophosphamide, and rituximab must be stopped for 2 weeks prior and 2 weeks after treatment. - Active pregnancy - Concurrent administration of any drugs with known radiosensitive effects (e.g. Methotrexate). |
| Study type | Interventional Allocation: non-randomized Masking: none  Assignment: single-arm  Purpose: outcome assessment Phase II |
| Date of first enrolment | October 2018 |
| Target sample size | 39 |
| Recruitment status | Recruiting |
| Primary outcome(s) | Overall survival [Time Frame: 4 years]: time from enrollment to death from any cause. |
| Key secondary outcomes | - Toxicity as measured by the Common Terminology Criteria for Adverse Events (CTCAE) version 4.0 [Time Frame: 8 years] - Progression-Free Survival [Time Frame: 8 years] - Local Control as determined via radiographic evidence [Time Frame: 8 years] - Cough Severity as reported by the participant via 10 cm analogue Cough Severity Scale [Time Frame: 8 years] - Rates of Acute-Exacerbation of Idiopathic Pulmonary Fibrosis (IPF) [Time Frame: 8 years] - Rates of Acute-Exacerbation of ILD [Time Frame: 8 years] - Quality of Life measured by the Functional Assessment of Cancer Therapy - Lung questionnaire [Time Frame: 8 years] - Changes in ILD Severity measured by High Resolution Computed Tomography (HRCT) [Time Frame: 8 years] - Changes in Pulmonary Function Tests [Time Frame: 8 years] - Exploratory Quantitative Analysis of High Resolution Computed Tomography (HRCT) Features [Time Frame: 8 years] - Analysis of Outcomes for Patients Eligible for Study Who Decline Radiotherapy [Time Frame: 8 years] - Quality of Life measured by the EuroQOL Group EQ-5D-5L questionnaire [Time Frame: 8 years] |
